# Supplementary figures and images for: Promiscuous structural cross-compatibilities between major shell components of Klebsiella pneumoniae bacterial microcompartments
Source: PLoS One. 2025 May 7;20(5):e0322518. doi: 10.1371/journal.pone.0322518 (PMC12058022; doi:10.1371/journal.pone.0322518)

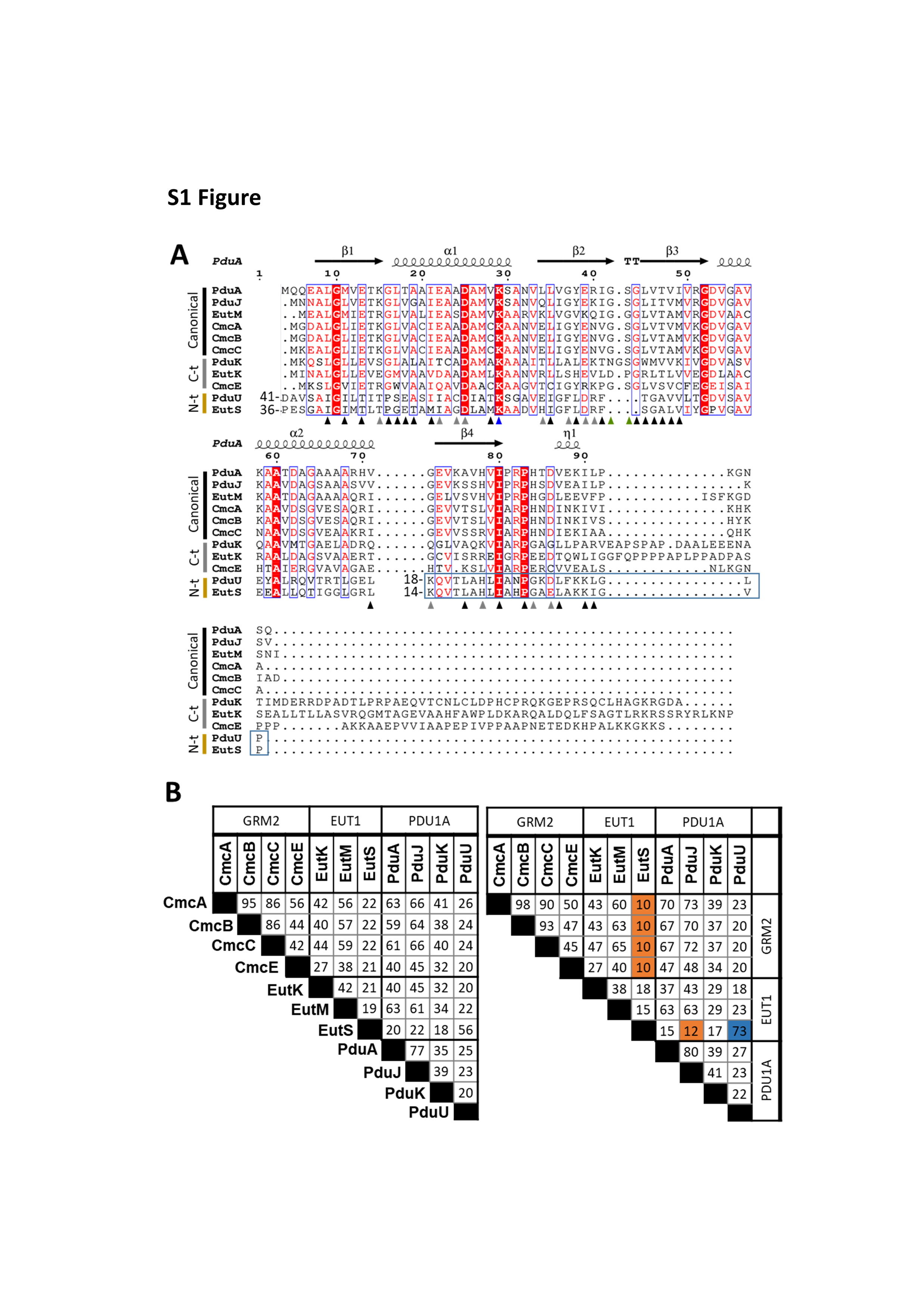

Supplement: S1 Fig — A. Sequence alignments were prepared with the RCSB pairwise alignment tool (TM-align method), taking as input the structures from the BMC-H monomers generated by AF2. Secondary structure elements from PduA are indicated as reference. EutS and PduU present secondary structure permutations. They were aligned to PduA using the JCE-CP (flexible) method and manually verified. The permutation is highlighted by the black rectangle, indicating the number of the first shown residue. PduU and EutS residues that build the N-terminal β-barrel were excluded. Residues fully or partly embedded at the interface between monomers are indicated by black or grey arrows, respectively. Critical lysine (K26 in PduA) and pore residues that also participate to the interface are indicated by blue and green arrows, respectively. The presentation was generated online with ESPript 3.0. B. Percentage of sequence identity between Kpe BMC-H. Values based on either residues belonging to the common BMC-H core domain (left) or only those falling at the interface between monomers (right). Coloured values are to highlight cases exhibiting more than 10% discrepancy of identity when comparing the two sets of residues. (TIF) [file pone.0322518.s001.tif]

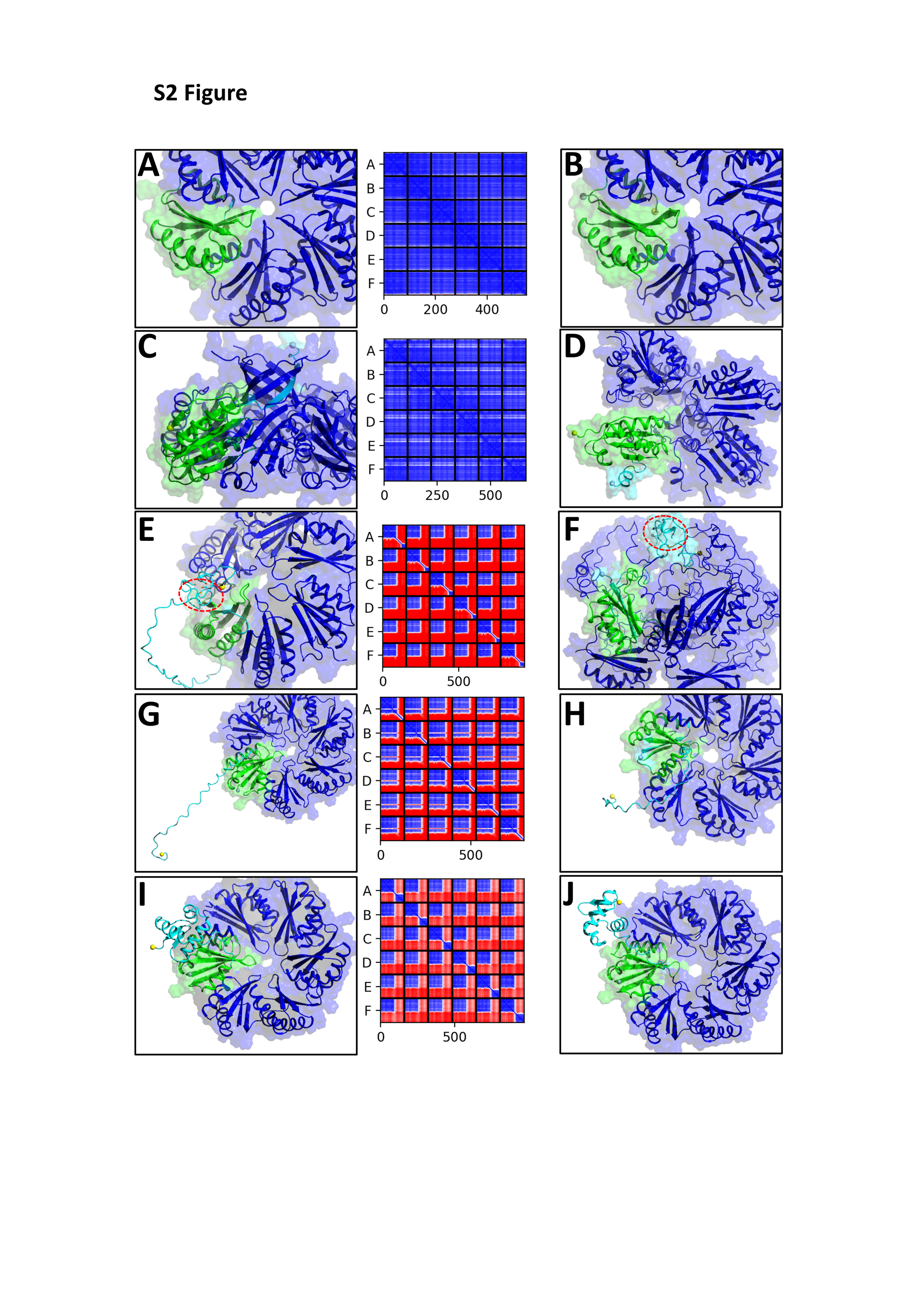

Supplement: S2 Fig — Cartoon illustration of portions of representative BMC-H structures predicted by AF2 (left panels) or ESMFold (right panels). From top to bottom are presented the next BMC-H: CmcA (panels A,B), EutS (C,D), PduK (E,F), CmcE (G,H) and EutK (I,J). The first monomer of the hexamer is colored green, all other monomers are in blue. Views are from the hexamer convex side for CmcA and PduK, concave side for CmcE and EutK. In the case of EutS, a side view is shown. Please note that ESMFold did not predict hexamer for EutS (D) and PduK (F). The cysteine-rich domain of PduK is highlighted by the dashed red circles, with S-atoms appearing as orange sticks. To simplify the views, C-terminal extensions in the last three rows are shown only for the first hexamer (cyan). Light green and yellow spheres indicate the localization of the N- or C-terminal residue of the first monomer, respectively. In the middle are included inter-residue pAE score matrices for AF2 structural predictions on the left. Coloring scheme is the one applied by the online Colab AF2 tool (e.g., deep blue for highest confidence level). A to E labels identify each monomer. For each monomer in diagonal elements of PduK or EutK matrices, a first blue box corresponding to BMC-H core residues is followed by small or moderate size blue boxes, respectively, thus supporting high confident folds. (TIF) [file pone.0322518.s002.tif]

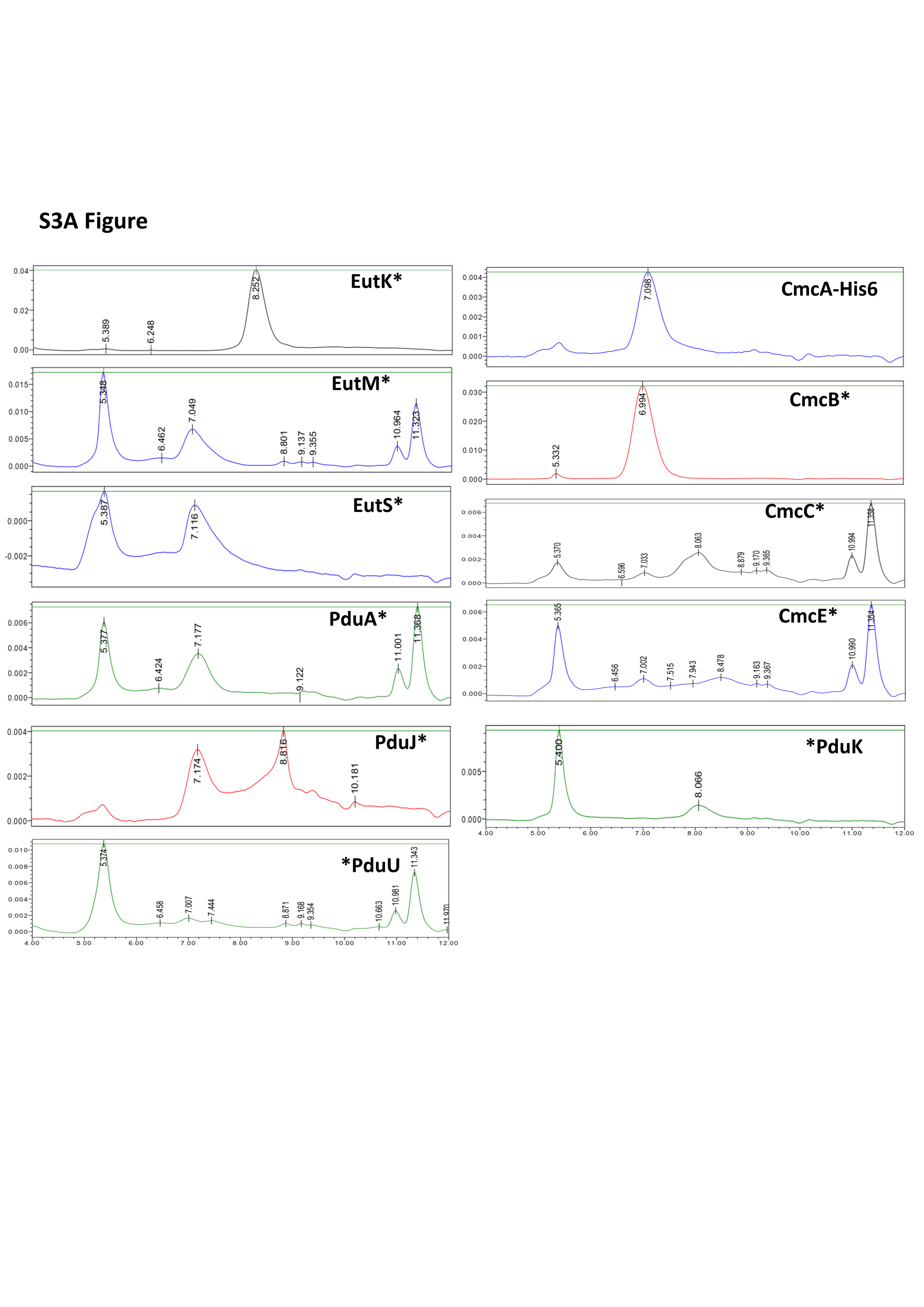

Supplement: S3A Fig — Profiles after injection of urea purified fractions from indicated BMC-H. EutM and CmcB chromatograms correspond to purified samples (before urea treatments). (TIF) [file pone.0322518.s003.tif]

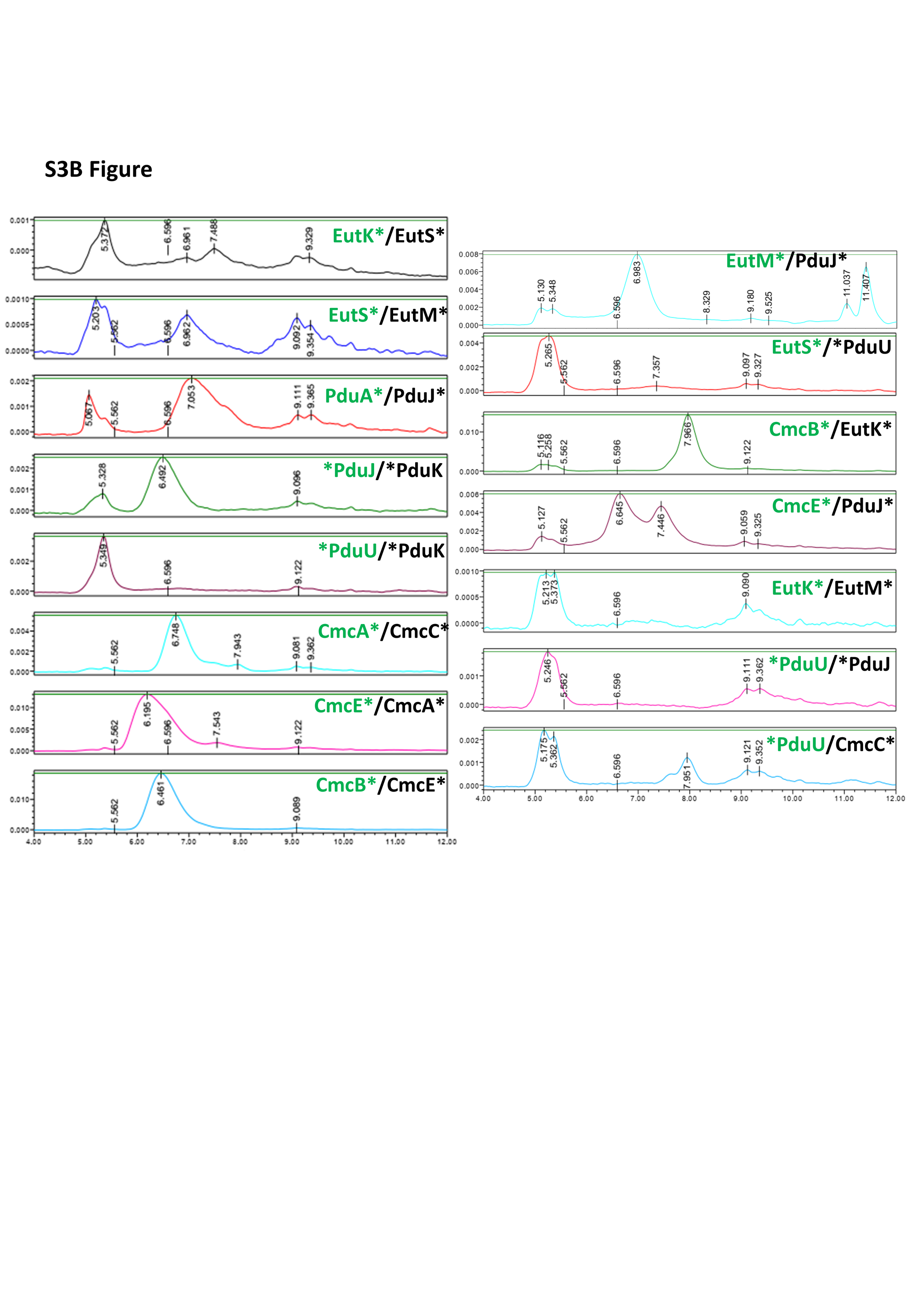

Supplement: S3B Fig — Chromatograms obtained after injection of purified fractions from strains co-expressing indicated BMC-H combinations. Black labels identify the His6-tagged component, in green for the FLAG-partner. N- or C-terminal tag localization is indicated by an asterisk preceding or following BMC-H identity, respectively. (TIF) [file pone.0322518.s004.tif]

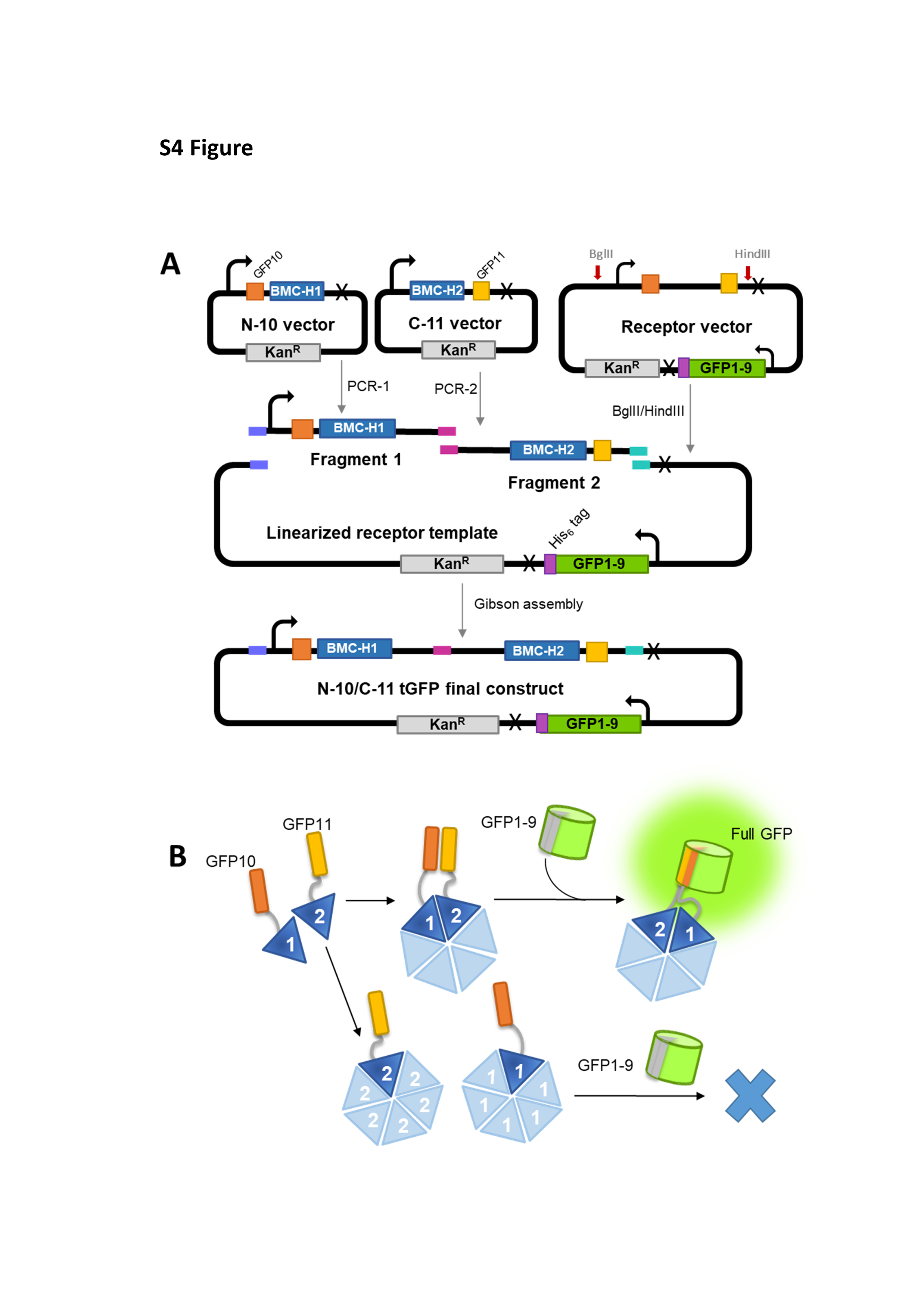

Supplement: S4 Fig — A. General strategy of construction of plasmids for tGFP assays. Preliminarily, plasmids coding for individual BMC-H with GFP10 or GFP11 tags in either C- or N-terminal were mounted on a pET26b-based template, giving rise to N-10, C-10, N-11 or C-11 vectors. Sequences coding for the two BMC-H of interest were then amplified by PCR from the corresponding plasmids. For simplicity, only one (N-10/C-11) of the eight possible combinations for a given pair of BMC-H is shown here. Primers included 15 nucleotide regions allowing hybridization to either the adjacent fragment (pink box) or to the receptor vector (blue and magenta). The tGFP receptor vector (KanR) included necessary information for the independent expression of the GFP1–9. The fragments and opened vector were Gibson-assembled giving rise to the final tGFP construct. T7 promoters and terminators are indicated by the arrows and crosses, respectively. B. tGFP assay principle: in the case of two interacting BMC-H, the GFP10 and 11 tags will come closer to each other. Reconstitution of a full fluorescent GFP will therefore be promoted in the presence of the GFP1–9 portion. Conversely, GFP reconstitution will be inefficient with non-interacting BMC-Hs. (TIF) [file pone.0322518.s005.tif]

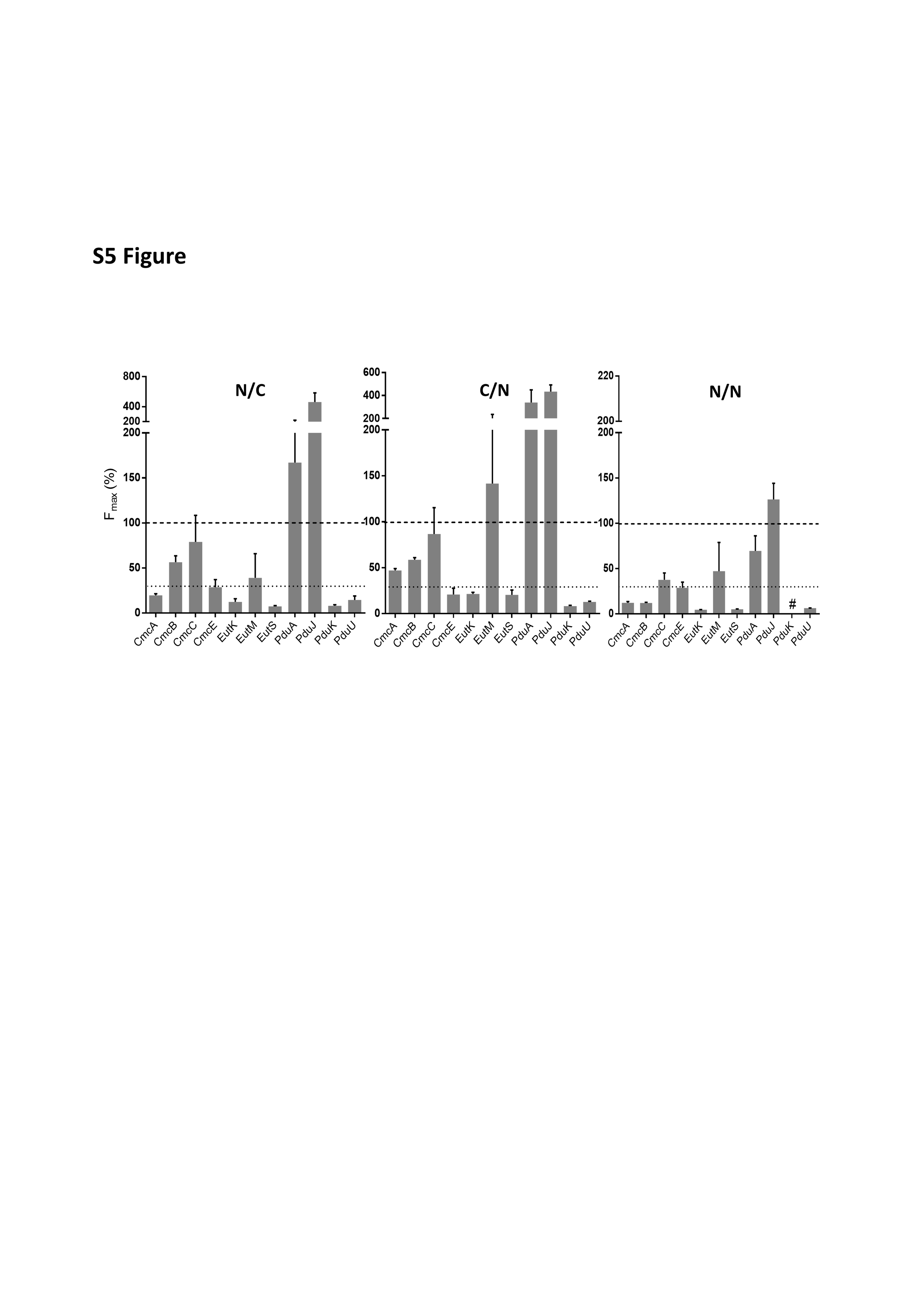

Supplement: S5 Fig — Fluorescence signals deriving from co-expression of Kpe BMC-H homo-pairs with GFP10/GFP11 tags attached following different configurations: N/C, C/N, or N/N orientations. The preparation of plasmid corresponding to the N/N PduK combination failed and could not be assayed (# symbol). Plotted mean Fmax and standard deviations derive from at least 2 independent repetitions of experiments, each one performed on 3 clones per library member. These values are provided in supplementary S1 File. Other experimental and data analysis details were the same as for Fig 5. (TIF) [file pone.0322518.s006.tif]

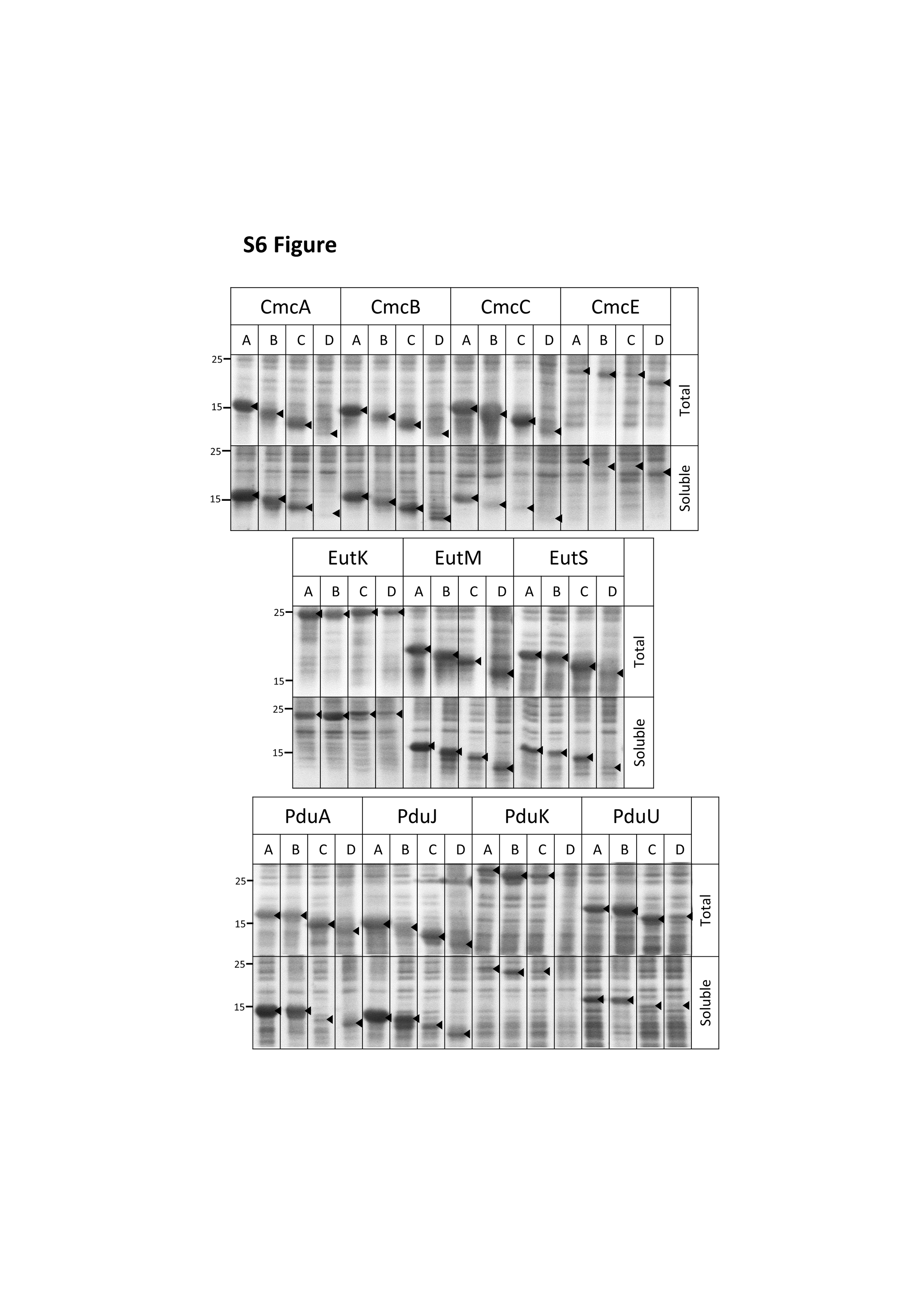

Supplement: S6 Fig — Individual BMC-H either tagged with GFP10 on C- (A) or N-terminus (B), or with GFP11 on the C- (C) or N-terminus (D) were over-expressed in BL21(DE3). After recovery of total cellular fractions, centrifugation permitted to prepare fractions corresponding to soluble contents. All fractions were analysed by SDS-PAGE and stained with Instant blue (Expedeon). The approximate migration of ladder components with indicated MW (kDa) are indicated on the left. Black arrows are given as an attempt to identify corresponding BMC-H bands. Theoretical MWs of BMC-H monomer constructs (kDa) are: CmcA 9.4; CmcB 9.6; CmcC 9.5; CmcE 13.6; EutK 17.0; EutM 9.8; EutS 11.6; PduA 9.8; PduJ 9.2; PduK 16.2; PduU 12.4. To these, it is necessary to add the contribution of linkers and tags, which differ as follows: for N-ter GFP10, 4.4; for C-ter GFP10, 4.6; for N-ter or C-ter GFP11, 4.7. (TIF) [file pone.0322518.s007.tif]

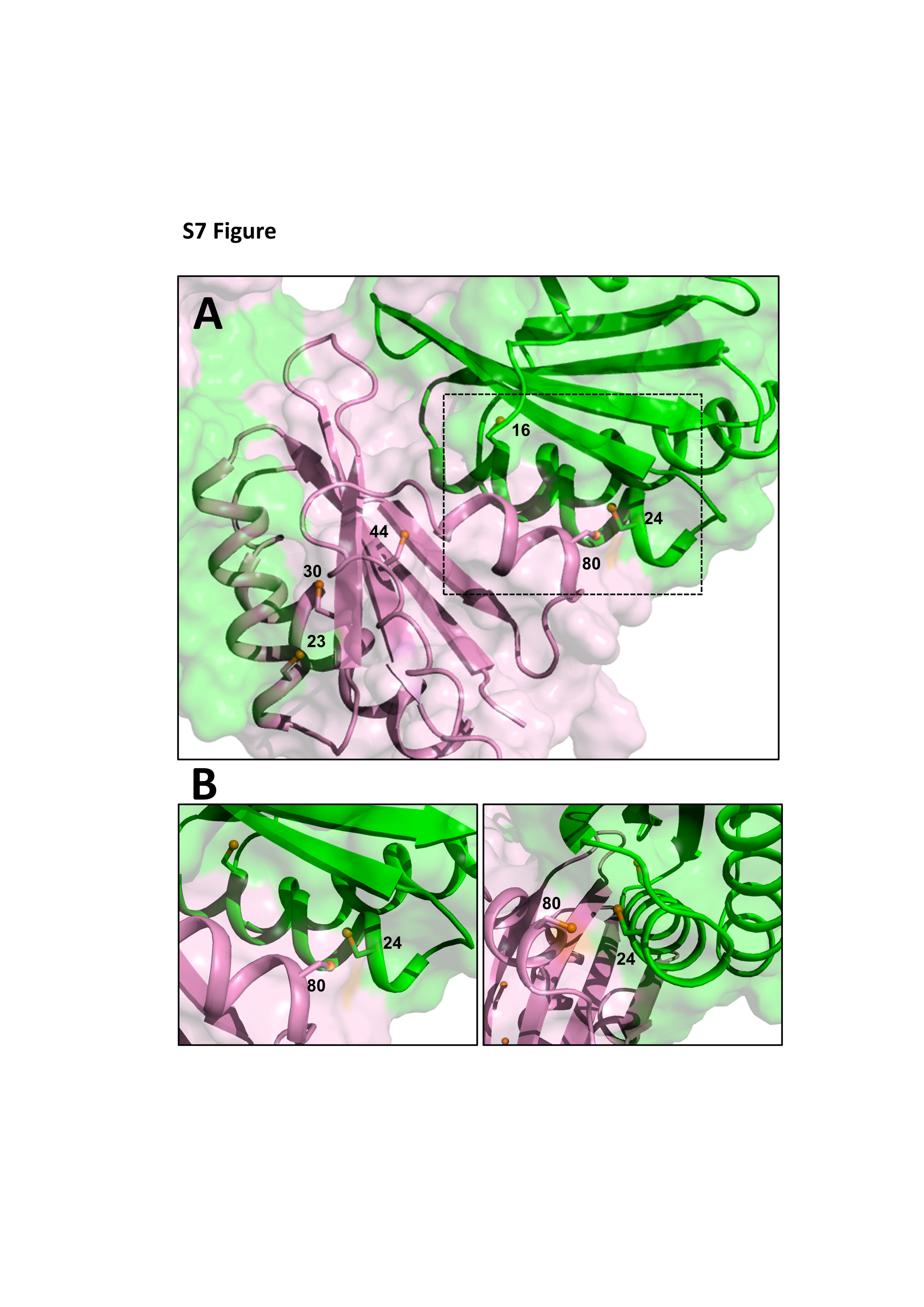

Supplement: S7 Fig — A, A potential disulfide bridge between residues Cys24 and Cys80 of CmcA (geen) and CmcE (violet), respectively, might stabilize contacts between monomers in hetero-hexameric associations. Depicted model corresponds to the AF2 prediction, but a similar result was obtained with ESMFold. Only two neighboring monomers are shown in cartoon representation to facilitate visualization. Cysteine side-chains are depicted as sticks with sulfur atoms as orange balls. Similar arrangement of cysteines is found when CmcA is replaced by CmcB or CmcC. B, magnified views of the region around CmcA24/CmcE80. (TIF) [file pone.0322518.s008.tif]

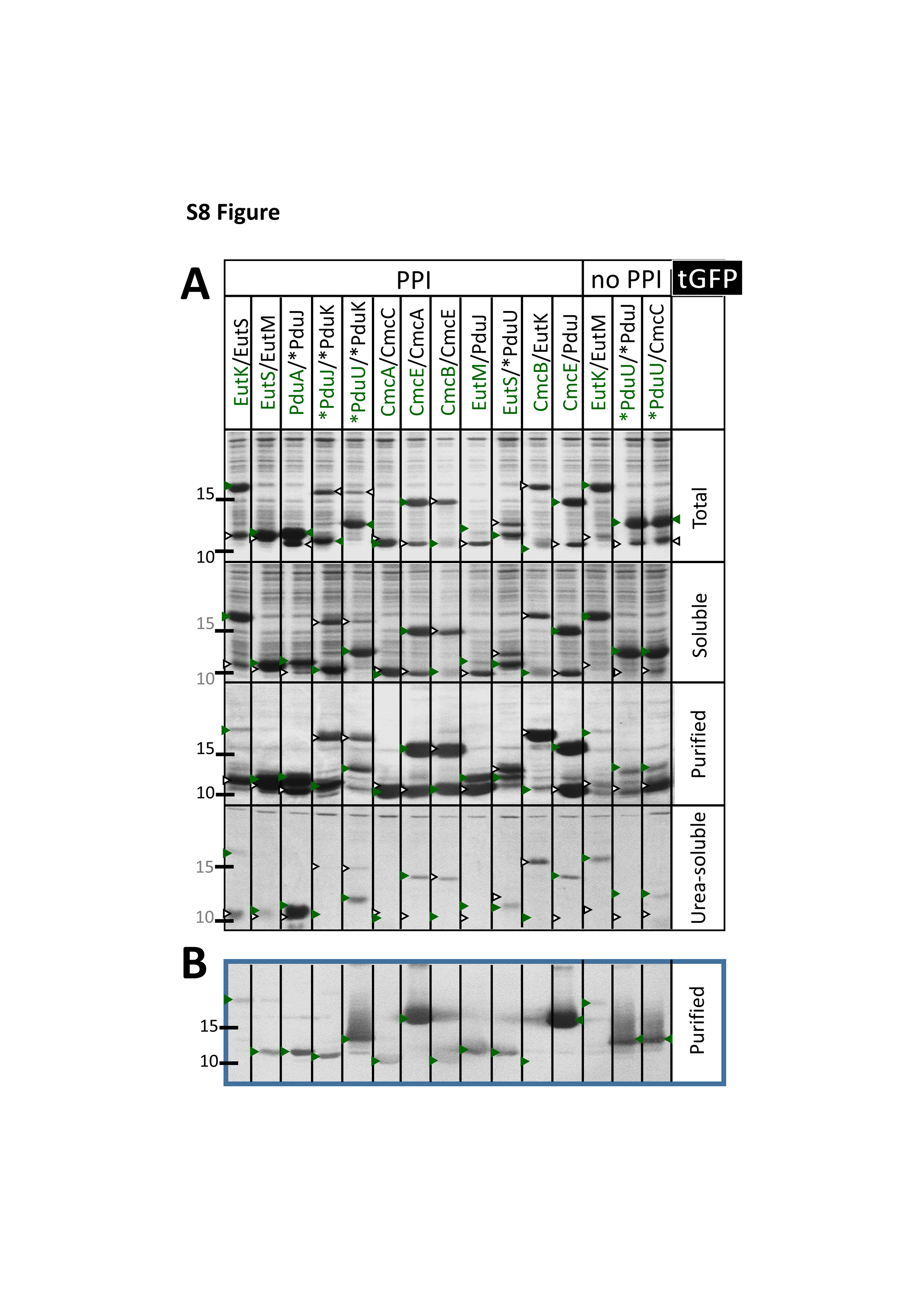

Supplement: S8 Fig — All details are as in Fig 6, with the exception that fractions were treated in the absence of β-mercaptoethanol. (TIF) [file pone.0322518.s009.tif]

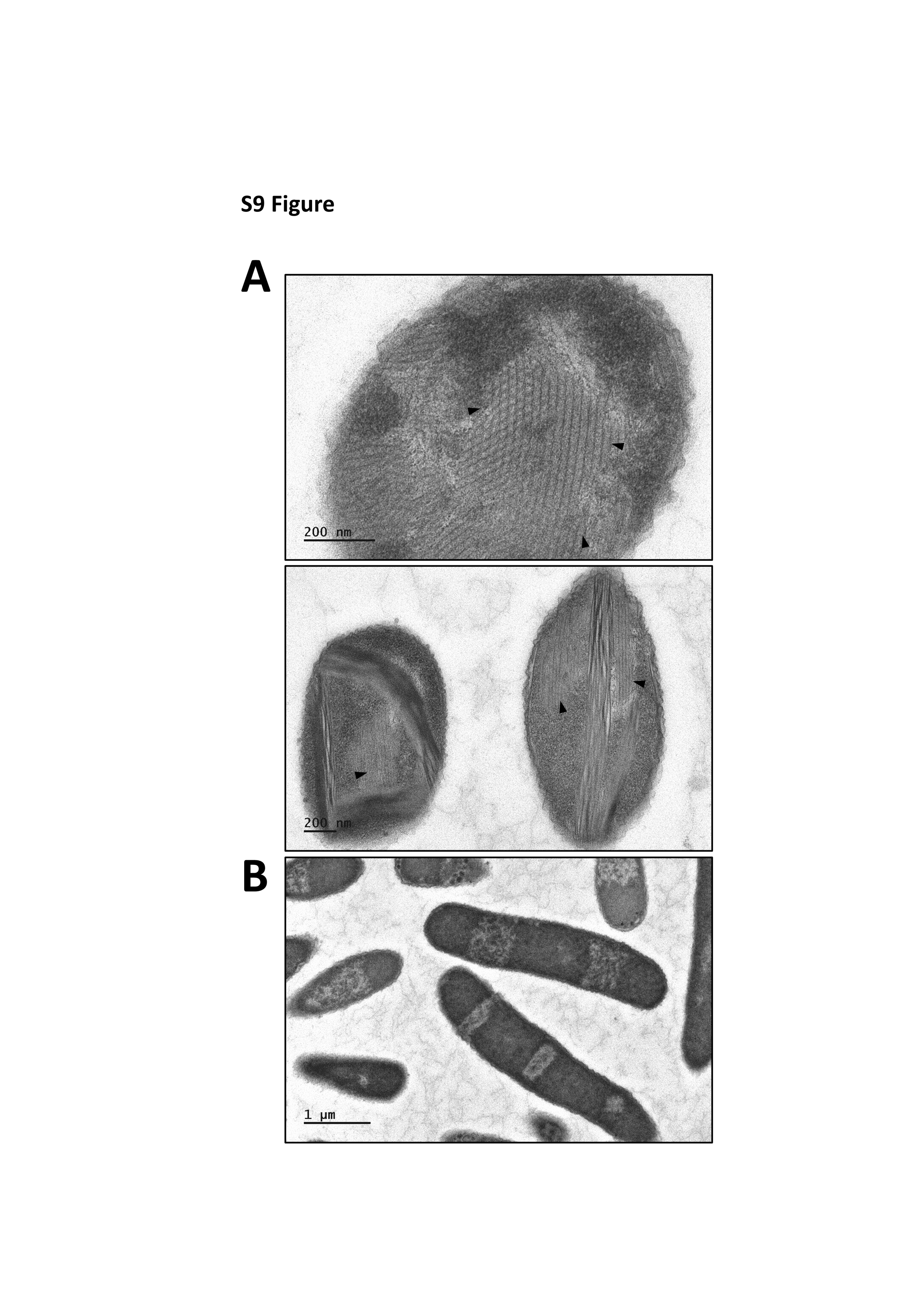

Supplement: S9 Fig — TEM images showing contents of E. coli cells after over-expression of hetero-pairs combining PduJ-FLAG/PduA-His6 (A) or CmcA-FLAG/CmcC-His6 (B). Regions displaying nanotubes are indicated by the arrows. (TIF) [file pone.0322518.s010.tif]

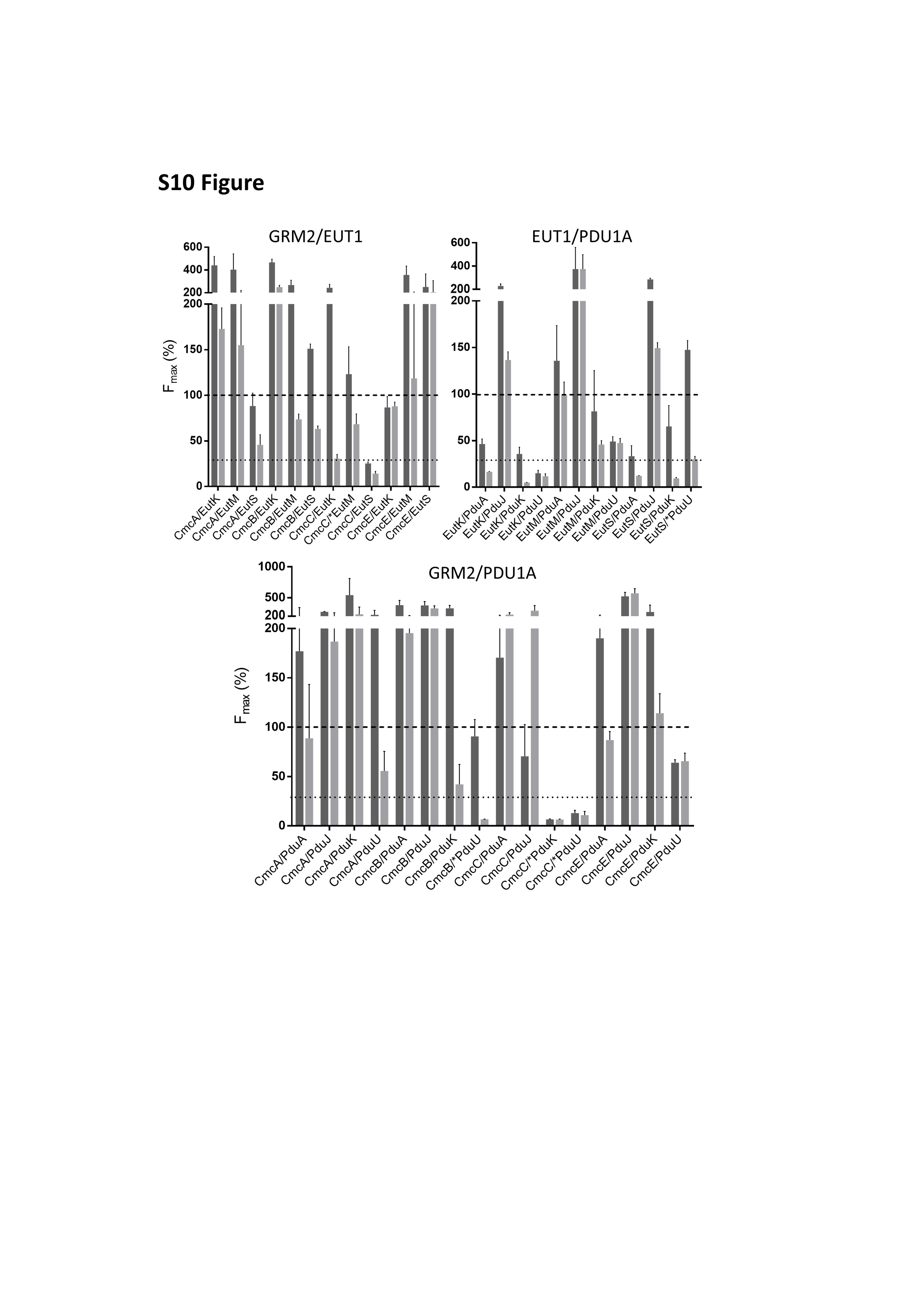

Supplement: S10 Fig — Please refer to Fig 5 for details on data presentation. (TIF) [file pone.0322518.s011.tif]

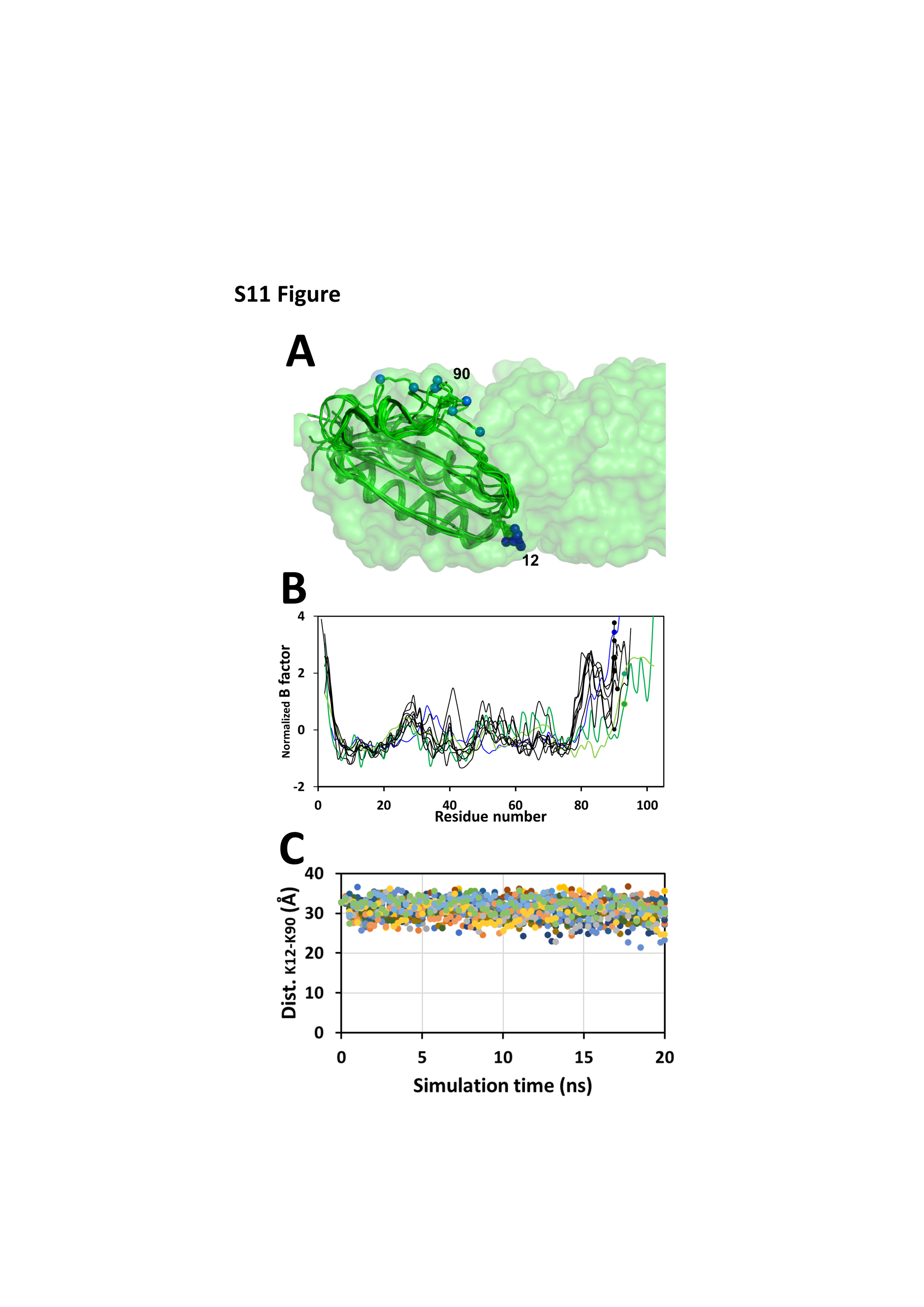

Supplement: S11 Fig — A. Comparison of the disposition of K12 and K90 side-chains in deposited 3D structures of PduA variants does not support big displacements towards the hexamer edge required to explain cross-links with K36 and K89 from neighboring PduJ hexamers. Instead, C-terminal residues (including K90) are most often modelled towards the central pore. Wild-type PduA (3NGK) is shown in black trace, in green for all mutants (4PPD, 4QIE, 4QIF, 4GIG, 4RBT, 4RBU, 4RBV). Terminal K12 and K90 amine groups appear as spheres. B. Comparison of normalized crystallographic B temperature factors for Cα atoms in the different structures. The blue trace is for 3NGK, black for PduA mutants and green for two CcmK2 structures discussed by Trettel et al. (4OX7 and 21AB)[41]. Values were averaged over equivalent modeled residues in the hexamer. Normalized B factor values seem indeed comparable between PduA K90 and the corresponding Arg93 of CcmK2 (dots, following the same color codes). The region preceding this position is not highly mobile, according to MD simulations discussed in ref [41]. C. Molecular dynamic analysis does not support K90 extensive movements. Plotted are the evolution of distances between K12/K90 side-chain amine atoms from each monomer during 20 ns run simulations of a PduA trihexameric assembly. Values never differed by more than 10 Å from the initial position, which is that from the crystal structure (3NGK). Shifts indicating approaches were due to movements towards the central pore. For each snapshot, 18 measurements are presented, corresponding to all the monomers from the 3NGK PduA tri-hexameric assembly studied before [58]. Note that inter-amine distances are measured in straight line, which is still incompatible with reaction in most instances. (TIF) [file pone.0322518.s012.tif]
