# Supplementary material for: Promiscuous structural cross-compatibilities between major shell components of Klebsiella pneumoniae bacterial microcompartments
Source: PLoS One. 2025 May 7;20(5):e0322518. doi: 10.1371/journal.pone.0322518 (PMC12058022; doi:10.1371/journal.pone.0322518)
Supplement: S1 Table — a Expressions values are calculated from three independent replicates, and are given as fold changes relative to the condition without substrate.b SEM values correspond to the expression lower and upper error bars. Control measurements of housekeeping gene expression are compiled in S2 Table. (PDF) [file pone.0322518.s013.pdf]

S1 Table. Induction of *Kpe* BMC transcription in response to metabolite presence.

Substrates	Exp.[a]	SEM[b]	P-value	Exp.[a]	SEM[b]	P-value	Exp.[a]	SEM[b]	P-value
	cmcA			***cutC***			***cmcE***		
w/o	1.0	0.8<br>1.3	/	1.0	0.9<br>1.1	/	1.0	0.9<br>1.1	/
EA	1.0	0.4<br>2.3	0.966	0.4	0.2<br>0.9	0.335	0.2	0.1<br>0.3	0.057
PD	0.4	0.2<br>0.7	0.219	2.4	1.7<br>3.5	0.077	0.6	0.4<br>0.9	0.266
CL	476.9	422.2<br>538.7	< 0.001	76.0	59.0<br>97.9	< 0.001	22.9	18.5<br>28.4	< 0.001
EA + PD	0.8	0.3<br>1.8	0.797	0.3	0.3<br>0.4	0.003	0.2	0.2<br>0.3	0.004
EA + CL	17.9	9.8<br>32.9	0.012	2.8	1.2<br>6.3	0.287	1.0	0.6<br>1.8	0.987
PD + CL	602.6	535.2<br>678.5	< 0.001	67.0	54.6<br>82.3	< 0.001	17.0	13.7<br>21.1	< 0.001
	eutS			***eutM***			***eutK***		
w/o	1.0	1.0<br>1.0	/	1.0	0.8<br>1.2	/	1.0	0.8<br>1.3	/
EA	1.1	0.8<br>1.4	0.831	3.8	3.2<br>4.4	0.006	0.4	0.3<br>0.5	0.083
PD	0.7	0.6<br>0.7	0.002	2.2	1.7<br>2.9	0.081	1.0	0.9<br>1.0	0.922
CL	0.7	0.6<br>0.8	0.054	17.1	15.0<br>19.5	< 0.001	1.3	1.2<br>1.4	0.422
EA + PD	0.6	0.5<br>0.6	0.005	9.2	6.7<br>12.6	0.004	0.3	0.3<br>0.3	0.015
EA + CL	13.1	2.7<br>63.7	0.178	477.8	96.5<br>2366.9	0.019	5.7	1.5<br>22.1	0.273
PD + CL	0.7	0.6<br>0.7	0.016	22.7	20.1<br>25.7	< 0.001	1.0	1.0<br>1.1	0.944
	pduA			***pduJ***			***pduU***		
w/o	1.0	0.8<br>1.3	/	1.0	0.7<br>1.5	/	1.0	0.8<br>1.2	/
EA	0.1	0.0<br>0.1	0.027	0.3	0.1<br>0.9	0.344	0.9	0.3<br>2.7	0.929
PD	0.8	0.7<br>1.0	0.516	3.8	2.4<br>5.9	0.095	5.7	3.0<br>10.7	0.059
CL	5.1	4.3<br>6.0	0.005	36.2	28.8<br>45.6	0.002	26.7	24.4<br>29.2	< 0.001
EA + PD	1.5	1.0<br>2.2	0.437	0.4	0.4<br>0.5	0.139	0.8	0.5<br>1.2	0.641
EA + CL	0.6	0.5<br>0.6	0.106	2.7	2.0<br>3.6	0.117	1.7	1.1<br>2.5	0.32
PD + CL	19.0	17.1<br>21.0	< 0.001	78.7	63.9<br>96.9	< 0.001	47.4	36.8<br>61.2	< 0.001

[a] Expressions values are calculated from three independent replicates, and are given as fold changes relative to the condition without substrate.

[b] SEM values correspond to the expression lower and upper error bars.

Control measurements of housekeeping gene expression are compiled in S2 Table.
