# Supplementary material for: Promiscuous structural cross-compatibilities between major shell components of Klebsiella pneumoniae bacterial microcompartments
Source: PLoS One. 2025 May 7;20(5):e0322518. doi: 10.1371/journal.pone.0322518 (PMC12058022; doi:10.1371/journal.pone.0322518)
Supplement: S2 Table — a mean quantification cycle, determined from three technical replicates. (PDF) [file pone.0322518.s014.pdf]

S2 Table. Reproducibility of transcription level measurements of housekeeping genes.

Target genes	Substrates	Mean Cq[a]
proC	w/o	28.38
		27.64
		27.63
	EA	26.16
		25.00
		23.86
	PD	26.29
		25.88
		26.37
	CL	24.67
		24.25
		24.18
	EA + PD	25.38
		25.98
		26.53
	EA + CL	24.55
		24.57
		25.32
	PD + CL	24.87
		24.82
		25.14

Target genes	Substrates	Mean Cq[a]
rpoD	w/o	27.24
		26.60
		26.53
	EA	24.66
		23.81
		23.19
	PD	28.26
		27.29
		27.76
	CL	24.59
		24.29
		24.46
	EA + PD	24.48
		25.23
		25.54
	EA + CL	24.45
		24.99
		24.63
	PD + CL	25.11
		25.02
		25.00

[a] mean quantification cycle, determined from three technical replicates.
