# Supplementary material for: Promiscuous structural cross-compatibilities between major shell components of Klebsiella pneumoniae bacterial microcompartments
Source: PLoS One. 2025 May 7;20(5):e0322518. doi: 10.1371/journal.pone.0322518 (PMC12058022; doi:10.1371/journal.pone.0322518)
Supplement: S3 Table — a Successful prediction of PF00936 hexameric associations is specified as YES/NO. b Global pLDDT. c interchain PAE, between pairs of residues belonging to different chains. d Values between pairs of interchain residues lying closer than 4 Å from each other. e values for interchain residues belonging to the BMC-H core, only calculated for non-canonical BMC-H. f AF-multimer pTM and ipTM scores below 0.7 indicate low confidence prediction quality. g Interaction energy (ΔE) computed using Rosetta InterfaceAnalyzer after structure relaxation, and averaged over all interfaceΔs. Values of if_pLDDT, if_PAE and ΔE are reported only for combinations predicted as PF00936 hexamers. (PDF) [file pone.0322518.s015.pdf]

S3 Table. Analysis of ESMFold and AF2 predictions for homo-hexamers

Protein	Hex[a]	pLDDT[b]	ic_PAE[c]	Interface[d]		Core[e]	pTM[f]	ipTM[f]	ΔE[g]
				pLDDT	PAE	c_PAE			
**AlphaFold2**									
**CmcA**	YES	95.8	1.7	96.6	1.4	-	0.95	0.94	-63.5
**CmcB**	YES	95.6	1.8	95.2	1.6	-	0.94	0.94	-58.6
**CmcC**	YES	94.9	1.9	95.6	1.5	-	0.94	0.94	-54.9
**EutM**	YES	95.4	1.9	94.8	3.4	-	0.94	0.94	-80.3
**PduA**	YES	93.4	2.3	95.6	3.1	-	0.92	0.92	-62.9
**PduJ**	YES	94.5	2.0	95.1	1.6	-	0.93	0.92	-61.1
**CmcE**	YES	72.8	7.4	90.4	2.8	1.9	0.70	0.69	-54.4
**EutK**	YES	82.8	5.7	85.9	6.6	1.6	0.77	0.77	-80.2
**PduK**	YES	67.7	7.7	73.1	9.5	3.0	0.65	0.63	-51.3
**EutS**	YES	96.1	1.7	95.8	3.0	1.4	0.94	0.93	-70.1
**PduU**	YES	92.6	2.5	95.6	1.6	1.8	0.92	0.92	-77.2
**BWI**	NO	76.2	7.5	-	-	-	0.45	0.36	-
**CcmK3**	YES	96.2	1.8	96.3	1.5	-	0.93	0.93	-72.0
**RMM**	YES	93.6	2.3	96.6	2.8	-	0.92	0.91	-71.6
**ESMFold**									
**CmcA**	YES	80.1	2.9	86.4	1.9	-			-54.3
**CmcB**	YES	78.9	3.1	84.0	2.0	-			-61.7
**CmcC**	YES	81.9	2.6	87.4	1.7	-			-53.8
**EutM**	YES	75.9	3.4	84.7	1.8	-			-61.5
**PduA**	YES	76.8	3.4	80.6	2.7	-			-53.4
**PduJ**	YES	80.9	2.9	85.7	2.0	-			-54.7
**CmcE**	YES	59.2	8.5	71.6	7.8	4.2			-14.2
**EutK**	YES	64.7	9.3	79.5	3.0	3.0			-53.1
**PduK**	NO	47.1	12.3	-	-	9.3			-
**EutS**	NO	63.2	9.2	-	-	8.5			-
**PduU**	YES	62.8	5.2	64.6	7.3	4.5			-61.3
**BWI**	NO	69.8	12.0	-	-	-			-
**CcmK3**	NO	59.3	7.3	-	-	-			-
**RMM**	YES	78.1	3.3	83.7	2.2	-			-64.2

[a] Successful prediction of PF00936 hexameric associations is specified as YES/NO. [b] Global pLDDT. [c] interchain PAE, between pairs of residues belonging to different chains. [d] Values between pairs of interchain residues lying closer than 4 Å from each other. [e] values for interchain residues belonging to the BMC-H core, only calculated for non-canonical BMC-H. [f] AF-multimer pTM and ipTM scores below 0.7 indicate low confidence prediction quality. [g] Interaction energy (*ΔE*) computed using Rosetta InterfaceAnalyzer after structure relaxation, and averaged over all interfaces. Values of if_pLDDT, if_PAE and *ΔE* are reported only for combinations predicted as PF00936 hexamers.
