# Supplementary material for: Promiscuous structural cross-compatibilities between major shell components of Klebsiella pneumoniae bacterial microcompartments
Source: PLoS One. 2025 May 7;20(5):e0322518. doi: 10.1371/journal.pone.0322518 (PMC12058022; doi:10.1371/journal.pone.0322518)
Supplement: S4 Table — a Averaged over all interfaces present in the 3D structure. (PDF) [file pone.0322518.s016.pdf]

**S4 Table. Evaluation of inter-monomer interaction energies from experimental 3D structures**

| Name | RCSB | Origin | Commentary | ΔE[a] |
|---|---|---|---|---|
| BMC-H | 4QIV | *Aer. hydrophila* | | -59.0 |
| BMC-H | 4OLO | *Clos. bacterium* | apo form (Fe-S cluster) | -51.5 |
| BMC-H | 5DJB | *Hal. ochraceum* | | -65.0 |
| BMC-H | 6NLU | *Hal. ochraceum* | with circular permutation | -62.7 |
| CcmK1 | 3BN4 | *Syn. sp. PCC 6803* | | -77.5 |
| CcmK1 | 3DN9 | *Syn. sp. PCC 6803* | missing C-terminal residues | -69.6 |
| CcmK2 | 2A1B | *Syn. sp. PCC 6803* | | -66.9 |
| CcmK2 | 3CIM | *Syn. sp. PCC 6803* | missing C-terminal residues | -75.5 |
| CcmK2 | 3SSQ | *Ther. elongatus BP-1* | stacked hexamer | -86.7 |
| CcmK2 | 4OX7 | *Syn. elon. PCC 7942* | | -80.1 |
| CcmK2 | 3DNC | *Syn. sp. PCC 6803* | missing C-terminal residues | -75.5 |
| CcmK4 | 2A10 | *Syn. sp. PCC 6803* | | -82.6 |
| CcmK4 | 6SCR | *Syn. sp. PCC 6803* | | -80.7 |
| CcmK4 | 2A18 | *Syn. sp. PCC 6803* | | -80.1 |
| CcmK4 | 5VGU | *Hal. sp. PCC 7418* | | -62.8 |
| CcmK4 | 4OX6 | *Syn. elon. PCC 7942* | | -83.5 |
| CmcA | 7MGP | *E. coli* | K25A mutant | -65.1 |
| CmcB | 7MPW | *E. coli* | K25D mutant | -46.0 |
| CmcC | 7MPV | *E. coli* | K25A mutant | -57.2 |
| CsoS1 | 4OX8 | *Pro. marinus* | | -65.7 |
| CsoS1A | 2EWH | *Hal. neapolitanus* | | -69.5 |
| CsoS1A | 2G13 | *Hal. neapolitanus* | | -71.3 |
| CsoS1C | 3H8Y | *Hal. neapolitanus* | | -71.4 |
| CutN | 7MMX | *Strep. intermedius* | K27D mutant | -63.0 |
| CutR | 6XPI | *Strep. intermedius* | | -70.8 |
| CutR | 6XPK | *Strep. intermedius* | Screw-shaped hexamer | -35.1 |
| EutM | 3I6P | *E. coli* | | -72.3 |
| EutM | 3MPW | *E. coli* | | -66.3 |
| EutM | 3MPY | *E. coli* | | -73.1 |
| EutM | 4AXJ | *Clos. difficile* | | -63.7 |
| EutS | 3I96 | *E. coli* | dimer of trimers (ben hexamer) | -69.8 |
| EutS | 3IA0 | *E. coli* | G39V mutant, flat hexamer | -79.7 |
| PduA | 3NGK | *Sal. enterica* | | -56.0 |
| PduA | 4P7T | *Citr. freundii* | K26D mutant | -50.6 |
| PduA | 4P7V | *Citr. freundii* | K26D mutant | -56.6 |
| PduJ | 5D6V | *Sal. enterica* | Wild-type K25 manually reintroduced | -55.8 |
| PduU | 3CGI | *Sal. enterica* | | -76.4 |
| RMM | 5L38 | *Myc. Smegmatis* | | -69.1 |

[a] Averaged over all interfaces present in the 3D structure
