# Supplementary material for: Promiscuous structural cross-compatibilities between major shell components of Klebsiella pneumoniae bacterial microcompartments
Source: PLoS One. 2025 May 7;20(5):e0322518. doi: 10.1371/journal.pone.0322518 (PMC12058022; doi:10.1371/journal.pone.0322518)
Supplement: S5 Table — aTheoretical molecular weight (MW) of monomer, in kDa. b MW estimated from peak elution volume, in kDa. Labels 1-M and 2-M are to indicate first or second major species in intensity, whereas m is for minor or faintly detected species; ND for nothing detected within the 5–500 kDa resolving range of the column. c These samples revealed peaks eluting as high-MW soluble species of highest intensity (1-M). Their retention time was between those of ferritin (440kDa) and dextran blue (2MDa), or even above the latter. Asterisks indicate tag attachment to the BMC-H N-terminus. (PDF) [file pone.0322518.s017.pdf]

S5 Table –Assessment of *Kpe* BMC-H oligomeric state by SEC-HPLC.

	BMC-H$_{(His6)}$		
	BMC-H	MW[a]	SEC-MW[b]
EUT1	EutK	18.2	9/17 (1-M)
	EutM [c]	11.1	67 (2-M)
	EutS [c]	12.9	62 (2-M)
PDU1A	PduA	10.8	58 (1-M)
	PduJ	10.3	58 (2-M)<br>9 (1-M)
	*PduK [c]	17.6	22 (2-M)
	*PduU [c]	13.8	71 (m)
GRM2	CmcA	10.6	67 (1-M)
	CmcB	10.8	72 (1-M)
	CmcC	10.6	68 (m)<br>21 (1-M)
	CmcE [c]	14.7	71 (m)<br>13 (2-M)
	*CmcE [c]	15.0	71 (m)

BMC-H$_{(FLAG)}$/BMC-H$_{(His6)}$			
BMC-H	MW[a]	SEC-MW[b]	
EutK/EutS [c]	18.4/12.9	45 (2-M)	EUT1
EutS/EutM [c]	13.1/11.1	67 (2-M)	
PduA/*PduJ [c]	11.0/10.6	76 (1-M)	PDU1A
*PduJ/*PduK [c]	10.8/17.6	110 (1-M)	
*PduU/*PduK [c]	14.0/17.6	ND	
CmcA/CmcC	10.8/10.6	90 (1-M)<br>39 (2-M)	GRM2
CmcE/CmcA	14.9/10.6	153 (1-M)<br>34 (2-M)	
CmcB/CmcE	11.0/14.7	120 (1-M)	
EutM/PduJ	11.3/10.3	73 (1-M)	HYBRID
EutS/*PduU [c]	13.1/13.8	ND	
CmcB/EutK	11.0/18.2	12 (1-M)	
CmcE/PduJ	14.9/10.3	69 (1-M)<br>30 (2-M)	

[a]Theoretical molecular weight (MW) of monomer, in kDa. [b] MW estimated from peak elution volume, in kDa. Labels 1-M and 2-M are to indicate first or second major species in intensity, whereas m is for minor or faintly detected species; ND for nothing detected within the 5-500 kDa resolving range of the column. [c] These samples revealed peaks eluting as high-MW soluble species of highest intensity (1-M). Their retention time was between those of ferritin (440kDa) and dextran blue (2MDa), or even above the latter. Asterisks indicate tag attachment to the BMC-H N-terminus.
