# Supplementary material for: Promiscuous structural cross-compatibilities between major shell components of Klebsiella pneumoniae bacterial microcompartments
Source: PLoS One. 2025 May 7;20(5):e0322518. doi: 10.1371/journal.pone.0322518 (PMC12058022; doi:10.1371/journal.pone.0322518)
Supplement: S6 Table — a Organization mode of monomers in the top ranked predicted model. A and B are to identify the first and second monomer types. b Energies are averaged over the two different sets of 3 identical interfaces from the ABABAB hexamers. Other details are as in S3 Table. (PDF) [file pone.0322518.s018.pdf]

S6 Table. Analysis of ESMFold and AF2 predictions for hetero-hexamers combining monomers from the same BMC type.

BMC-H pair	Hex	Org.[a]	pLDDT	ic_PAE	Interface		Core	pTM	ipTM	ΔE[b]	
					pLDDT	PAE	c_PAE			A/B	B/A
**AlphaFold2**											
**CmcA/CmcB**	YES	ABABAB	94.4	2.0	94.8	3.8	-	0.94	0.93	-67.4	-63.7
**CmcA/CmcC**	YES	ABABAB	94.2	2.1	94.7	3.0	-	0.94	0.93	-62.6	-57.3
**CmcA/CmcE**	YES	ABABAB	81.2	5.5	85.8	8.7	4.6	0.79	0.81	-65.1	-60.0
**CmcB/CmcC**	YES	ABABAB	94	2.1	95.5	2.9	-	0.93	0.93	-66.8	-57.9
**CmcB/CmcE**	YES	ABABAB	81.2	5.4	85.6	8.7	4.4	0.79	0.80	-72.3	-63.3
**CmcC/CmcE**	YES	ABABAB	81.4	5.4	87	8.5	4.6	0.79	0.81	-65.1	-61.3
**EutK/EutM**	YES	ABABAB	88.2	5.8	91.4	11.5	4.8	0.76	0.78	-65.2	-89.0
**EutK/EutS**	YES	AAABBB	71.2	9.3	73.66	19.2	7.2	0.46	0.39	-	-
**EutM/EutS**	YES	AAABBB	83.4	5.4	74.9	12.4	5.2	0.68	0.63	-	-
**PduA/PduJ**	YES	ABABAB	93.5	2.2	94.9	3.9	-	0.92	0.92	-63.0	-63.1
**PduA/PduK**	YES	ABABAB	81.7	6.6	87	12.0	5.6	0.74	0.77	-63.0	-50.8
**PduA/PduU**	YES	AAABBB	79.4	6.6	78.4	15.8	6.3	0.61	0.55	-	-
**PduJ/PduK**	YES	ABABAB	82	6.5	85.9	12.9	5.6	0.74	0.77	-83.0	-46.7
**PduJ/PduU**	YES	AAABBB	79.1	6.8	77.5	16.6	6.5	0.59	0.52	-	-
**PduK/PduU**	YES	ABABAB	65.6	9.5	72.4	21.0	7.4	0.47	0.43	-26.2	-52.0
**CcmK1/CcmK2**	YES	ABABAB	95.4	2.0	96.6	3.5	2.0	0.94	0.94	-83.4	-82.4
**ESMFold**											
**CmcA/CmcB**	YES	ABABAB	80.24	2.9	85.0	2.1	-			-69.7	-74.2
**CmcA/CmcC**	YES	ABABAB	80.43	2.9	86.7	1.8	-			-58.7	-72.1
**CmcA/CmcE**	YES	ABABAB	67.62	5.0	81.0	2.6	3.3			-53.9	-64.3
**CmcB/CmcC**	YES	ABABAB	79.61	3.0	84.1	2.3	-			-66.7	-69.2
**CmcB/CmcE**	YES	ABABAB	68.63	4.9	81.3	2.6	3.4			-58.5	-63.9
**CmcC/CmcE**	YES	ABABAB	66.83	4.7	80.0	2.8	3.4			-55.9	-70.2
**EutK/EutM**	YES	ABABAB	70.87	7.2	80.8	2.8	3.3			-76.7	-81.4
**EutK/EutS**	YES	AAABBB	65.41	10.1	78.8	7.1	4.9			-	-
**EutM/EutS**	NO	-	72	8.5	-	-	8.0			-	-
**PduA/PduJ**	YES	ABABAB	80.45	2.9	85.7	2.0	-			-63.6	-69.5
**PduA/PduK**	YES	ABABAB	63.95	6.9	81.7	2.5	3.3			-52.7	-68.1
**PduA/PduU**	YES	AAABBB	67.01	6.4	71.5	14.2	5.7			-	-
**PduJ/PduK**	YES	ABABAB	64.55	6.9	82.8	2.4	3.2			-44.4	-61.9
**PduJ/PduU**	YES	AAABBB	68.57	5.8	75.7	9.8	5.1			-	-
**PduK/PduU**	YES	AAABBB	52.45	8.6	59.1	18.0	6.0			-	-
**CcmK1/CcmK2**	YES	ABABAB	71.2	4.3	80.9	2.5	3.5			-84.3	-81.0

[a] Organization mode of monomers in the top ranked predicted model. A and B are to identify the first and second monomer types. [b] Energies are averaged over the two different sets of 3 identical interfaces from the ABABAB hexamers. The last line corresponds to structural predictions for combined CcmK1 and CcmK2 from *Syn6803*. Other details are as in S3 Table.
