# Supplementary material for: Promiscuous structural cross-compatibilities between major shell components of Klebsiella pneumoniae bacterial microcompartments
Source: PLoS One. 2025 May 7;20(5):e0322518. doi: 10.1371/journal.pone.0322518 (PMC12058022; doi:10.1371/journal.pone.0322518)
Supplement: S7A Table — Data were organized as in S6 Table. (PDF) [file pone.0322518.s019.pdf]

S7A Table - Analysis of AF2 predictions for hetero-hexamers combining monomers from different BMC types. Data were organized as in S6 Table.

BMC-H pair	AlphaFold2										
	Hex	Org.	pLDDT	ic_PAE	Interface		Core	pTM	ipTM	ΔE	
					pLDDT	PAE	c_PAE			A/B	B/A
CmcA/EutK	YES	ABABAB	85.8	6.1	84.5	13.1	5.2	0.74	0.76	-71.4	-63.4
CmcA/EutM	YES	ABABAB	95.2	1.9	96.0	2.9	-	0.95	0.94	-70.2	-70.4
CmcA/EutS	YES	AAABBB	84.3	4.8	80.1	9.9	4.6	0.72	0.68	-	-
CmcB/EutK	YES	ABABAB	86.3	6.2	83.8	13.7	5.2	0.73	0.76	-74.2	-63.4
CmcB/EutM	YES	ABABAB	94.6	2.0	95.8	2.9	-	0.95	0.94	-71.0	-69.5
CmcB/EutS	YES	AAABBB	82.2	5.5	78.9	14.3	3.8	0.67	0.61	-	-
CmcC/EutK	YES	ABABAB	85	5.8	83.5	12.7	4.9	0.74	0.76	-79.4	-64.5
CmcC/EutM	YES	ABABAB	94.2	2.0	95.0	3.2	-	0.94	0.94	-62.6	-70.1
CmcC/EutS	YES	AAABBB	84.2	4.7	80.0	9.5	9.4	0.73	0.68	-	-
CmcE/EutK	YES	ABABAB	76.4	8.1	82.1	12.3	4.0	0.65	0.65	-53.5	-85.5
CmcE/EutM	YES	ABABAB	83.9	4.9	84.2	12.2	4.0	0.81	0.83	-71.5	-69.8
CmcE/EutS	YES	AAABBB	61.2	10.4	72.6	20.4	9.4	0.41	0.31	-	-
CmcA/PduA	YES	ABABAB	94.2	2.1	95.6	2.9	-	0.93	0.93	-62.3	-59.1
CmcA/PduJ	YES	ABABAB	94.8	2.0	96.1	2.8	-	0.94	0.93	-68.3	-62.5
CmcA/PduK	YES	ABABAB	82.4	6.4	86.8	12.2	5.5	0.75	0.77	-75.5	-49.5
CmcA/PduU	YES	AAABBB	80.4	6.3	85.4	13.3	5.9	0.61	0.55	-	-
CmcB/PduA	YES	ABABAB	93.8	2.2	95.7	3.4	-	0.93	0.92	-67.2	-65.1
CmcB/PduJ	YES	ABABAB	95.2	1.9	96.6	2.7	-	0.94	0.93	-67.3	-64.2
CmcB/PduK	YES	ABABAB	82.1	6.5	86.1	12.3	5.5	0.74	0.77	-71.1	-56.1
CmcB/PduU	YES	AAABBB	81.4	5.9	75.1	12.3	5.2	0.65	0.59	-	-
CmcC/PduA	YES	ABABAB	93.2	2.2	93.9	3.1	-	0.93	0.92	-59.2	-59.5
CmcC/PduJ	YES	ABABAB	94.4	2.0	95.1	3.5	-	0.93	0.93	-59.2	-63.6
CmcC/PduK	YES	ABABAB	82.1	6.5	85.8	12.3	5.5	0.74	0.77	-62.2	-48.7
CmcC/PduU	YES	AAABBB	79.2	6.7	73.9	15.6	5.5	0.59	0.53	-	-
CmcE/PduA	YES	ABABAB	81.3	5.5	80.5	14.1	4.5	0.79	0.80	-51.3	-61.0
CmcE/PduJ	YES	ABABAB	81.4	5.4	79.4	13.4	4.5	0.80	0.81	-56.4	-59.2
CmcE/PduK	YES	ABABAB	72.2	8.1	79.1	11.3	4.0	0.65	0.65	-72.1	-46.6
CmcE/PduU	YES	AAABBB	62.3	10.1	65.1	18.9	8.6	0.43	0.34	-	-
EutK/PduA	YES	ABABAB	86.1	6.3	85.8	12.3	5.3	0.73	0.75	-61.7	-77.6
EutK/PduJ	YES	ABABAB	87.7	4.6	87.4	10.1	3.9	0.81	0.83	-66.3	-78.3
EutK/PduK	YES	ABABAB	74.2	8.3	84.8	7.2	2.7	0.64	0.63	-68.0	-47.9
EutK/PduU	YES	AAABBB	68.8	10.0	57.5	18.2	7.9	0.42	0.34	-	-
EutM/PduA	YES	ABABAB	93.5	2.3	93.9	3.4	-	0.93	0.93	-70.0	-65.5
EutM/PduJ	YES	ABABAB	94.6	2.0	95.2	3.5	-	0.94	0.93	-69.9	-63.4
EutM/PduK	YES	ABABAB	80.9	6.5	82.6	13.7	5.3	0.75	0.77	-82.0	-50.5
EutM/PduU	YES	AAABBB	77.6	8.0	67.7	18.3	7.4	0.52	0.44	-	-
EutS/PduA	YES	AAABBB	81.4	5.7	76.6	11.9	5.3	0.67	0.62	-	-
EutS/PduJ	YES	AAABBB	80.2	6.5	74.8	15.5	6.2	0.61	0.54	-	-
EutS/PduK	YES	ABABAB	65.8	9.4	70.8	21.8	7.6	0.45	0.42	-51.9	-24.2
EutS/PduU	YES	ABABAB	94.1	2.2	93.6	5.5	1.7	0.93	0.93	-	-
CcmK1/CcmK2	YES	ABABAB	95.4	2.0	96.6	3.5	-	0.94	0.94	-83.4	-82.4
