# Supplementary material for: Promiscuous structural cross-compatibilities between major shell components of Klebsiella pneumoniae bacterial microcompartments
Source: PLoS One. 2025 May 7;20(5):e0322518. doi: 10.1371/journal.pone.0322518 (PMC12058022; doi:10.1371/journal.pone.0322518)
Supplement: S7B Table — Please refer to S6 Table for data organization details. (PDF) [file pone.0322518.s020.pdf]

S7B Table - Analysis of ESMFold predictions for hetero-hexamers combining monomers from different BMC types. Please refer to S6 Table for data organization details.

BMC-H pair	ESMFold								
	Hex	Org.	pLDDT	ic_PAE	Interface		Core	ΔE	
					pLDDT	PAE	c_PAE	A/B	B/A
CmcA/EutK	YES	ABABAB	70.8	7.2	82.6	2.6	3.1	-62.3	-70.0
CmcA/EutM	YES	ABABAB	80.3	3.0	87.42	1.74	-	-71.6	-74.7
CmcA/EutS	NO	-	74.8	8.5	-	-	8.1	-	-
CmcB/EutK	YES	ABABAB	69.6	7.3	81.8	2.7	3.3	-64.5	-70.4
CmcB/EutM	YES	ABABAB	78.5	3.2	85.22	1.96	-	-71.5	-81.8
CmcB/EutS	NO	-	73.7	8.5	-	-	8.1	-	-
CmcC/EutK	YES	ABABAB	71.0	7.3	81.8	2.5	3.0	-56.0	-69.9
CmcC/EutM	YES	ABABAB	79.9	2.9	86.55	1.86	-	-71.1	-61.6
CmcC/EutS	NO	-	74.0	8.4	-	-	8.0	-	-
CmcE/EutK	YES	ABABAB	64.0	8.7	78.6	3.5	3.4	-83.6	-49.4
CmcE/EutM	YES	ABABAB	65.0	6.0	77.0	3.6	3.7	-71.9	-76.2
CmcE/EutS	YES	AAABBB	57.0	8.2	75.4	15.4	6.9	-	-
CmcA/PduA	YES	ABABAB	78.7	3.1	83.3	2.26	-	-63.6	-78.7
CmcA/PduJ	YES	ABABAB	81.6	2.8	85.93	1.99	-	-61.1	-68.6
CmcA/PduK	YES	ABABAB	63.6	7.0	82.1	2.4	3.5	-50.1	-63.9
CmcA/PduU	NO	-	68.7	6.6	-	-	5.9	-	-
CmcB/PduA	YES	ABABAB	78.2	3.2	83.95	2.15	-	-68.0	-71.5
CmcB/PduJ	YES	ABABAB	80.3	3.0	86.11	1.9	-	-60.3	-71.0
CmcB/PduK	YES	ABABAB	63.8	5.9	79.5	3.0	3.5	-53.6	-59.7
CmcB/PduU	NO	-	68.2	6.5	-	-	5.8	-	-
CmcC/PduA	YES	ABABAB	80.2	2.9	85.37	1.93	-	-65.1	-65.9
CmcC/PduJ	YES	ABABAB	81.8	2.7	86.59	1.85	-	-59.7	-64.0
CmcC/PduK	YES	ABABAB	64.2	6.9	81.9	2.5	3.2	-48.5	-54.7
CmcC/PduU	YES	AAABBB	68.8	6.6	76.42	13.16	5.9	-	-
CmcE/PduA	YES	ABABAB	65.8	5.9	79.9	2.8	3.4	-60.8	-52.1
CmcE/PduJ	YES	ABABAB	67.1	5.4	78.5	3.3	3.3	-72.2	-56.2
CmcE/PduK	YES	ABABAB	59.5	8.4	75.9	4.9	3.7	-34.0	-37.4
CmcE/PduU	YES	AAABBB	53.6	9.2	68.5	15.7	7.0	-	-
EutK/PduA	YES	ABABAB	68.4	7.2	77.9	3.3	3.5	-70.2	-67.5
EutK/PduJ	YES	ABABAB	70.3	7.1	80.2	2.9	3.2	-66.8	-66.0
EutK/PduK	YES	ABABAB	59.8	9.3	77.2	4.0	3.4	-56.3	-79.3
EutK/PduU	YES	AAABBB	63.2	10.0	74.3	7.0	4.5	-	-
EutM/PduA	YES	ABABAB	78.4	3.2	84.41	2.23	-	-63.8	-71.4
EutM/PduJ	YES	ABABAB	79.7	3.0	85.38	2.04	-	-61.0	-72.5
EutM/PduK	YES	ABABAB	64.1	7.1	81.3	2.7	3.4	-69.2	-65.2
EutM/PduU	YES	AAABBB	65.5	6.5	68.62	14.22	5.8	-	-
EutS/PduA	NO	-	72.0	7.2	-	-	6.8	-	-
EutS/PduJ	NO	-	72.0	8.3	-	-	7.9	-	-
EutS/PduK	YES	AAABBB	55.3	8.5	75.6	13.4	7.1	-	-
EutS/PduU	YES	ABBAAB	68.4	4.9	77.97	3.66	3.4	-	-
CcmK1/CcmK2	YES	ABABAB	71.2	4.3	80.9	2.5	-	-84.3	-81.0
