# Supplementary material for: Promiscuous structural cross-compatibilities between major shell components of Klebsiella pneumoniae bacterial microcompartments
Source: PLoS One. 2025 May 7;20(5):e0322518. doi: 10.1371/journal.pone.0322518 (PMC12058022; doi:10.1371/journal.pone.0322518)
Supplement: S8 Table — (PDF) [file pone.0322518.s021.pdf]

S8 Table. Primers used in RT-qPCR experiments

Primer name	Primer sequence (5'- 3')	Use	Amplicon size (bp)
qPCR-eutS-Fw<br>qPCR-eutS-Rv	TCACACTGGCGCATCTGATT<br>CCCGGTGTCAGCGTCATAAT	Quantification of *eutS* expression by RT-qPCR	100
qPCR-eutM-Fw<br>qPCR-eutM-Rv	CGTATCGGTGAGCTGGTCTC<br>GCTATCGCCCTTGAAGCTGA	Quantification of *eutM* expression by RT-qPCR	90
qPCR-eutK-Fw<br>qPCR-eutK-Rv	TCCGGAAGAGGATACCCAGT<br>TAACGCTTCCGATGATGCCG	Quantification of *eutK* expression by RT-qPCR	94
qPCR-cmcA-Fw<br>qPCR-cmcA-Rv	GATGTGTAAAGCCGCCAACG<br>GACGTCGCCTTTCACCATCA	Quantification of *cmcA* expression by RT-qPCR	85
qPCR-cutC-Fw<br>qPCR-cutC-Rv	TTGACGGCTATCCGATGCTC<br>AACATGGCGGAGAGTTCGTT	Quantification of *cutC* expression by RT-qPCR	82
qPCR-cmcE-Fw<br>qPCR-cmcE-Rv	CATCCACACCGCCATTGAAC<br>CTTCAACCACACAGCGCTCC	Quantification of *cmcE* expression by RT-qPCR	98
qPCR-pduA-Fw<br>qPCR-pduA-Rv	AGGCTTAACCGCAGCCATAG<br>AACCGATCCTTTCGTAGCCC	Quantification of *pdA* expression by RT-qPCR	83
qPCR-pduJ-Fw<br>qPCR-pduJ-Rv	TTGAAGCCGCTGATGCAATG<br>GCGGACCATCACGGTAATCA	Quantification of *pduJ* expression by RT-qPCR	92
qPCR-pduU-Fw<br>qPCR-pduU-Rv	GCATTCTCACCATTACCCCCA<br>AAGCGATCGAGAAAGCCGAT	Quantification of *pduU* expression by RT-qPCR	94
qPCR-proC-Fw<br>qPCR-proC-Rv	GATTGCCGATATCGTCTTCG<br>GAGACCACCAGCGACTCTTT	Quantification of *proC* expression by RT-qPCR	99
qPCR-recA-Fw<br>qPCR-recA-Rv	TTAAACAGGCCGAATTCCAG<br>CCGCTTTCTCAATCAGCTTC	Quantification of *recA* expression by RT-qPCR	99
