# Supplementary material for: Promiscuous structural cross-compatibilities between major shell components of Klebsiella pneumoniae bacterial microcompartments
Source: PLoS One. 2025 May 7;20(5):e0322518. doi: 10.1371/journal.pone.0322518 (PMC12058022; doi:10.1371/journal.pone.0322518)
Supplement: S1_raw_images — Original uncropped and unadjusted images underlying SDS-PAGE gels and blots presented in this work. (PDF) [file pone.0322518.s025.pdf]

Raw data used for preparation of Figure 2 :

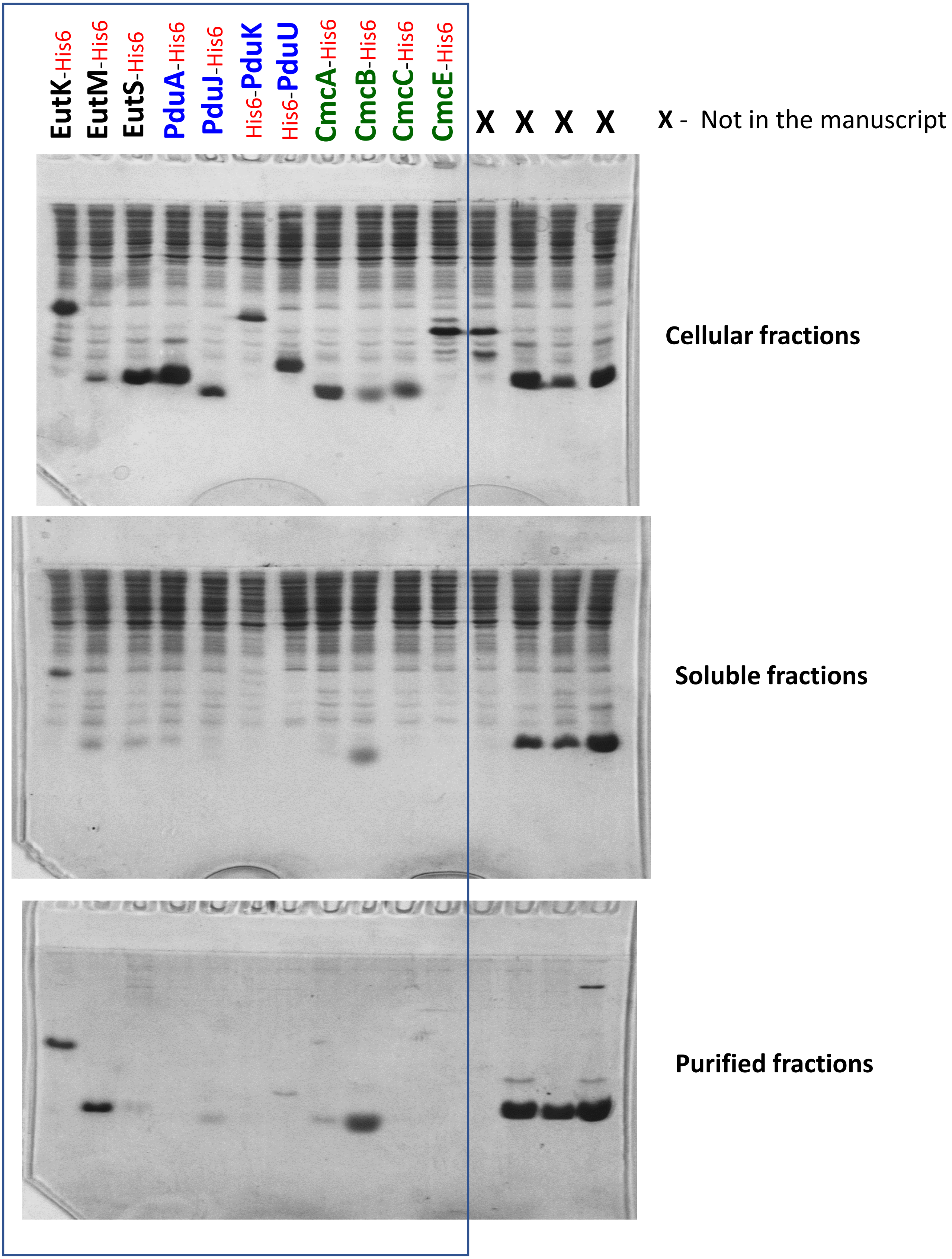

Data shown in the manuscript

Raw data used for preparation of Figure 2 (continuation)

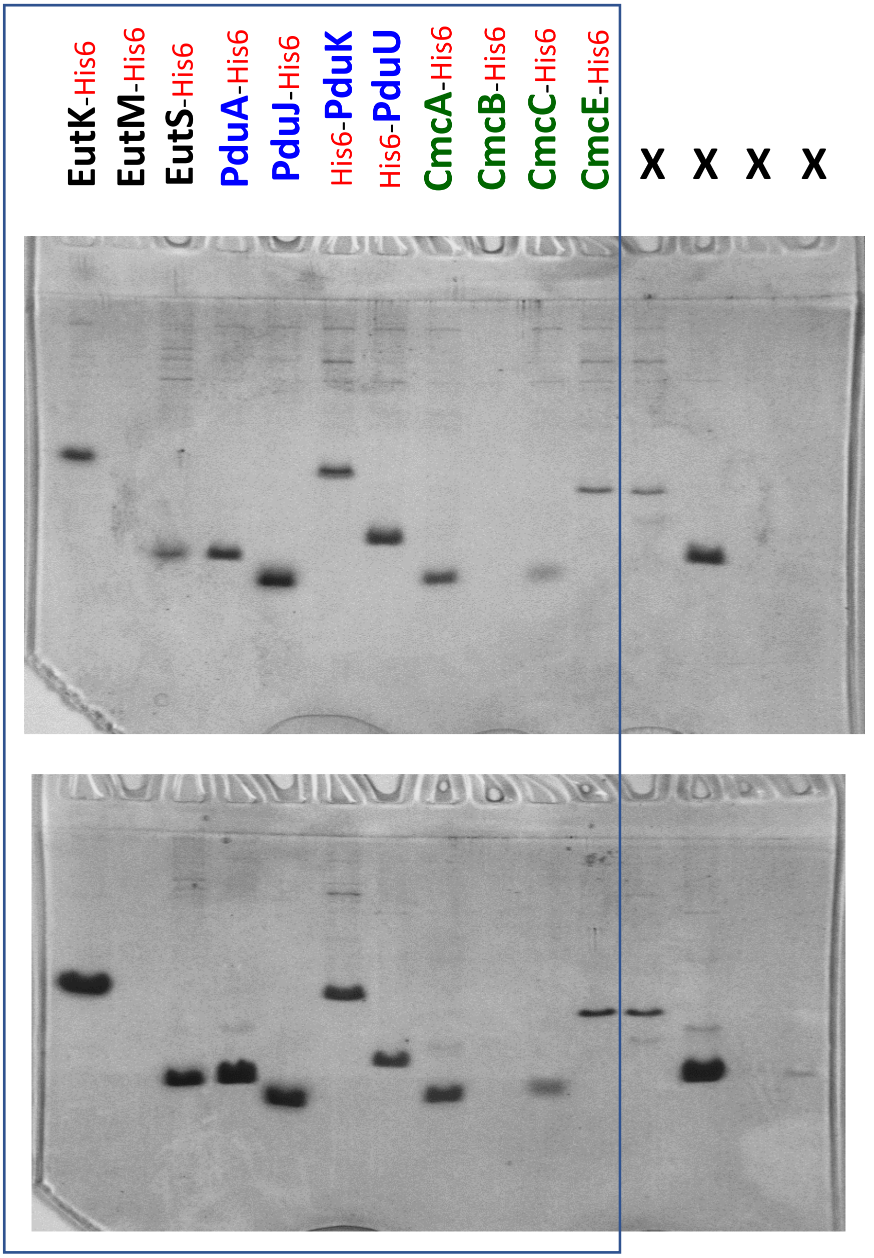

Raw data used for preparation of Figure 6 :

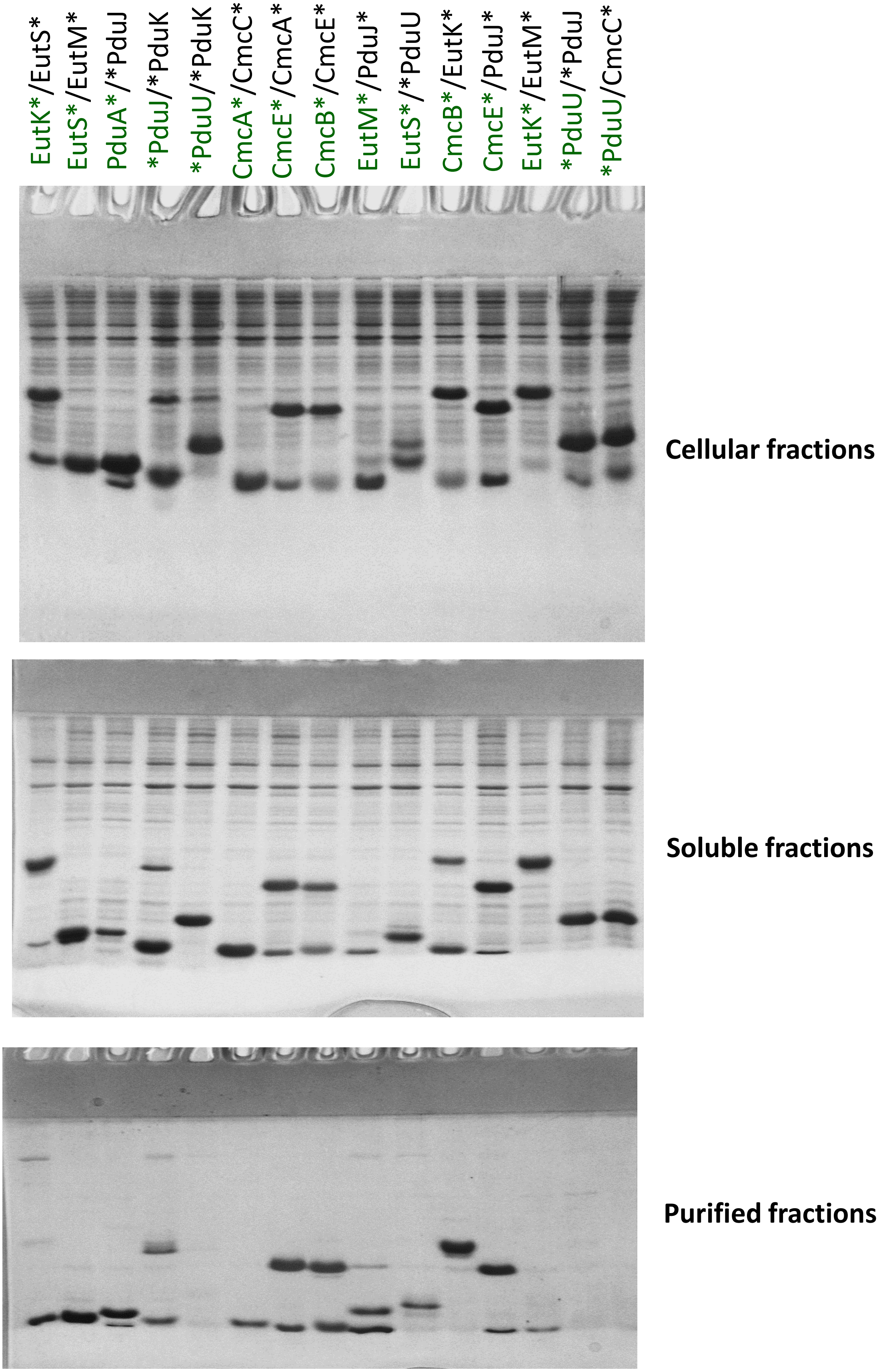

Raw data used for preparation of Figure 6 (continuation) :

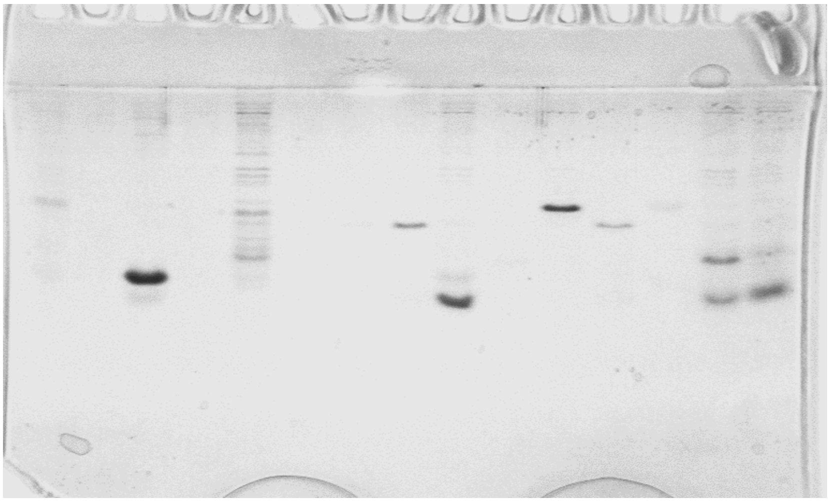

Urea solubilized fractions

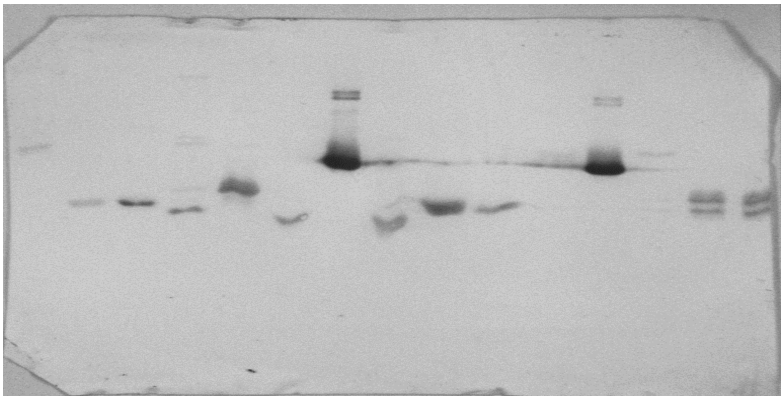

Top western blot, panel B

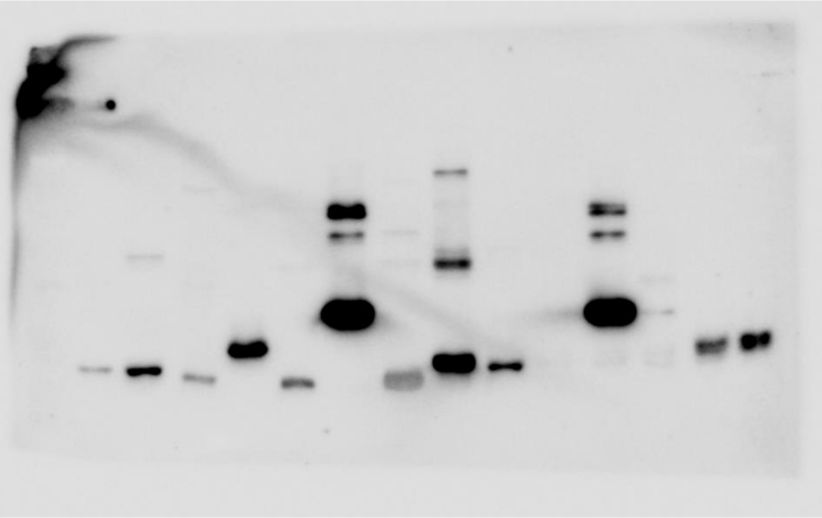

Bottom western blot, panel B

Raw data used for preparation of Figure S8 :

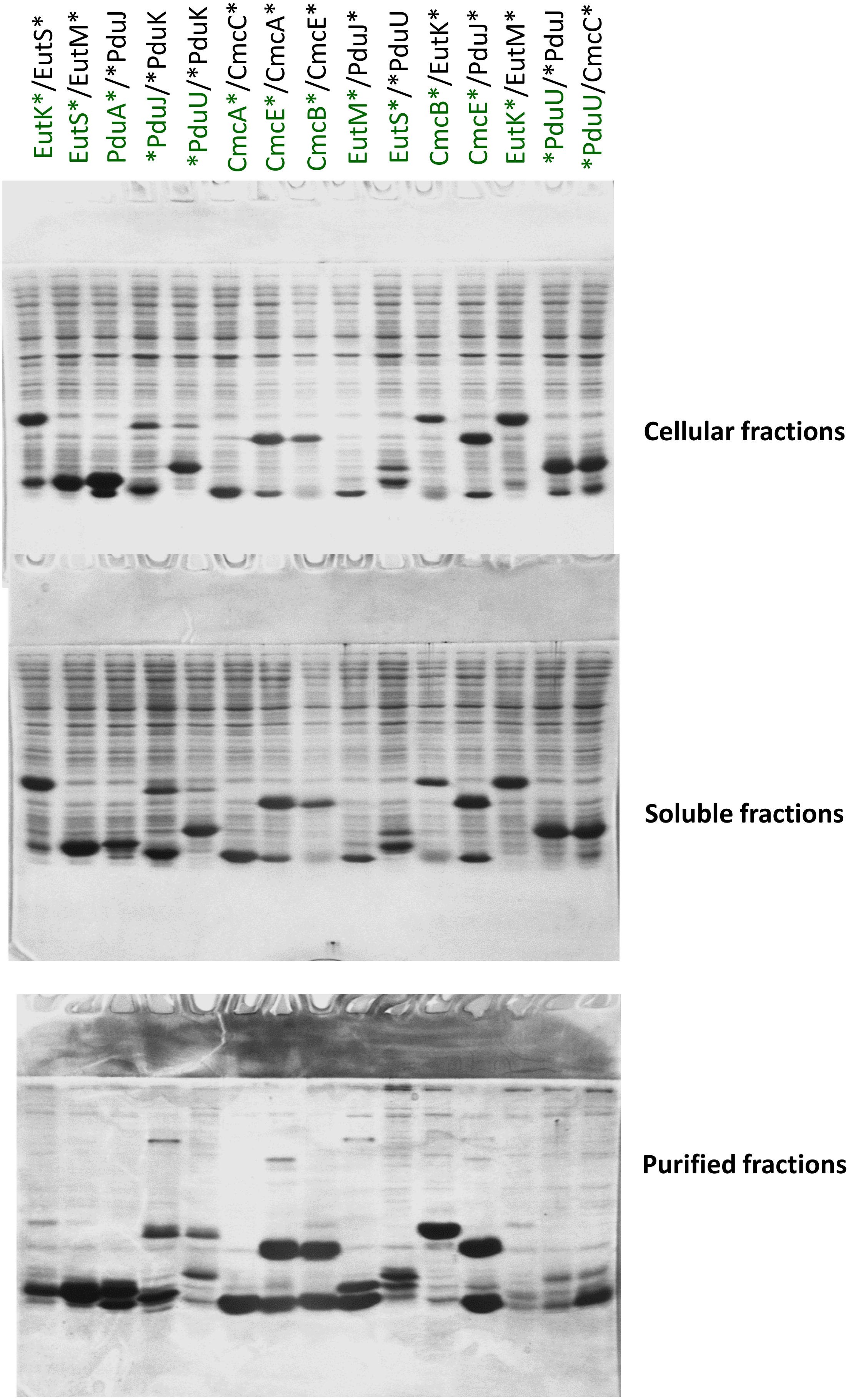

Raw data used for preparation of Figure S8 (continuation) :

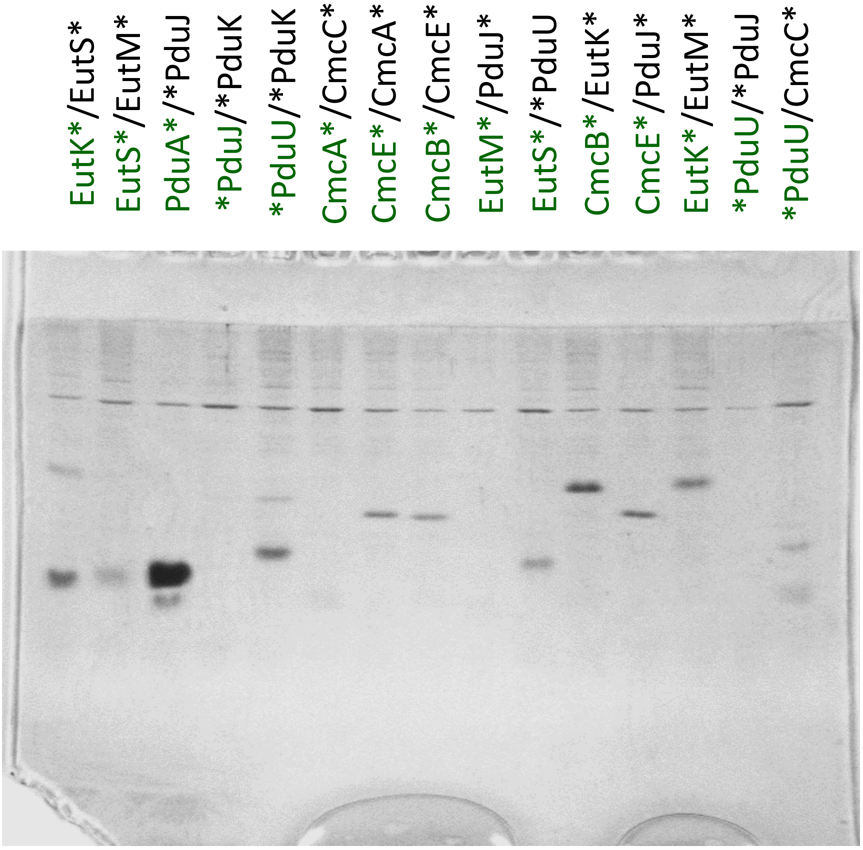

Urea solubilized fractions

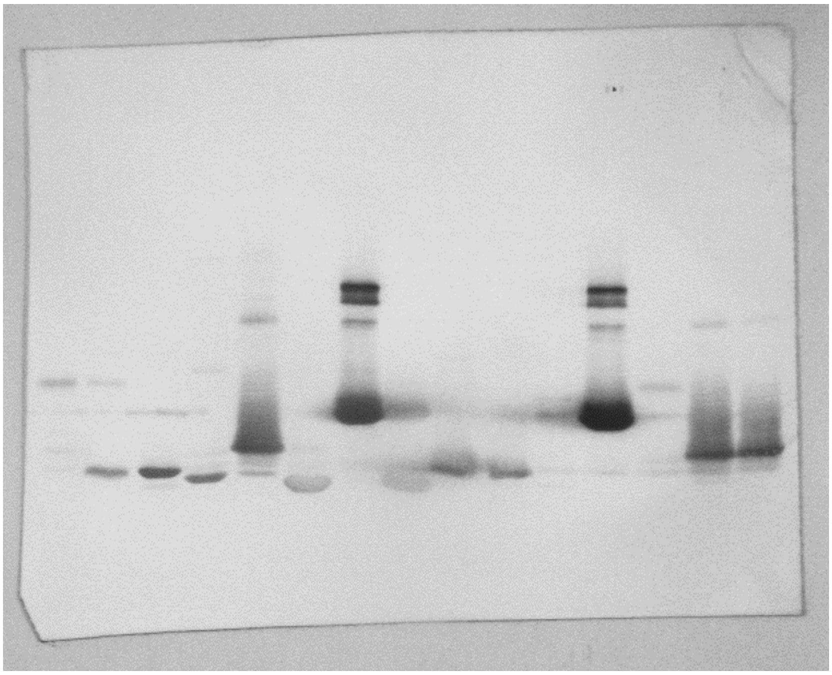

Western blot, panel B
