# Supplementary material for: Promiscuous structural cross-compatibilities between major shell components of Klebsiella pneumoniae bacterial microcompartments
Source: PLoS One. 2025 May 7;20(5):e0322518. doi: 10.1371/journal.pone.0322518 (PMC12058022; doi:10.1371/journal.pone.0322518)
Supplement: Attachment — Submitted filename: Response to reviewers.docx [file pone.0322518.s027.docx]

# Toulouse Biotechnology Institute

## UMR CNRS 5504 – UMR INRA 792

Toulouse, Tuesday, March 18, 2025

Please find below in black letters our reply to reviewers’ comments. Other minor changes were introduced, which are mentioned throughout this letter and can also be easily visualized in the “Revised Manuscript with Track Changes” version.

*Reviewer #1: I cannot review this work by the deadline given, as the formatting for what I was given for review requires too much flipping back and forth to see what I'm looking at, and the SI figures are missing captions altogether in the powerpoint given to the reviewers. The formatting chosen by the authors separates captions from figures, both in the main text and the SI. The main text figures themselves are of low quality, as though they had been through a lossy compression algorithm before I ever got to see them. I cannot make a determination on the work to see whether the conclusions (which seem reasonable based on the text!) are actually supported by the data, as the figures without their captions are insufficient for me to judge what is actually going on. The work itself is *probably* fine, and the conclusions again seem perfectly reasonable based on what I know about BMB and protein structure, but I can't verify the claims easily.*

We sincerely regret that Reviewer #1 suffered from such difficulties, and we apologize if that was our fault. In fact, it is unclear to us what was the cause. Though it is true that the pdf document generated automatically by the journal during submission was somehow disappointing, when comparing to the original images, the figures were still sufficiently clear in our hands, possibly for reviewers 2 and 3 too.

*Reviewer #2: This manuscript describes bioinformatic structural predictions of hetero-hexamer formation by bacterial microcompartment shell proteins from Klebsiella pneumoniae and goes on to co-expression studies demonstrating their likely formation in vitro by expression of different labelled protein pairs in E.coli. Because K.pneumoniae contains three different bacterial microcompartment operons there are a large number of possible combinations addressed. While all operons show some inter-operon protein associations, the Cut operon and Pdu operon shell proteins associate more than the Eut operon shell proteins do with proteins from either of the other two operons. There is some RT-PCR regulatory evidence that choline plus another substrate is more of a signal than 1,2-PD or ethanolamine. PduA/PduJ association is particularly favoured in the expression study and the structural prediction is of alternating monomers. The authors use this prediction to explain otherwise puzzling observations of a previous crosslinking study of BMC proteins from a heterologously expressed Salmonella Pdu operon by Trettel at al (1).


It is of interest to apply the bioinformatic tools to the problem of multiple BMC structures in the same organism, and the expression study correlations are useful.

I have the following points:

1. I don't think the application of their findings to Trettel et al' s observations is well expressed. I can't reconcile Figure 7 with Trettel Figure 6D: in Trettel 6D K90-K12 in the homo-hexame PduA/PduJ cross-linked structure is <25 angstroms, in Figure 7A it is >50: K86-K89 is > 30 angstroms in Trettel 6D, 22 Angstroms in Figure 7A. I think Figure 7 needs to be revised to make it clearer how the cross-linking data fits a heterohexamer PduA/PduJ than the Trettel PduA/PduJ homohexamer arrangement*

We agree with the reviewer, these data were somehow ambiguous in the original version and needed to be better explained. We tried to clarify things in the new version. However, there are some issues which are difficult to alter, as we will try to explain.

In fact, the apparent conflict was partially deriving from the fact that we provided distances between pairs of lysine side-chain nitrogen atoms (the reactive ones), whereas alpha carbons might have been the choice in Trettel’s study. However, we are not confident in stating anything with regard to their study, as we failed to find indications in this regard within their manuscript.

A second contribution to the discrepancy comes from the fact that providing the distance between two atoms in straight-line is somehow misleading. Other chemical entities positioned between the two groups would prevent the reaction. This is the case between K90 and K12 of PduA, which lie on opposite sides of the hexamer. That said, when considering a straight-line, the distance between Cα atoms of these two residues is <25 Å (i.e. 20.1 Å for the closest residues of a PduA 3NGK hexamer). The value becomes 29 Å when taking the reactive side-chain N atoms. As we showed in Fig. S10 (now S11 Fig), conformational flexibility might bring such reactive amines a bit closer to each other. However, we opted by indicating > 50 Å to signify that, if K90 revolved towards a neighbor hexamer, as Trettel et al. hypothesized to explain reactions with a neighbor PduJ hexamer, the reactive probe would need to contour the protein surface to also reach the K12, as it is unreasonable to expect that such probe will pass through the protein core. On the other hand, the alternative pathway that we propose to explain the K12-K90 cross-link, with the probe going through the central hexamer “pore” of PduA, would not provide any argument to these authors justifying the reaction with PduJ residues.

With regard to the couple K86(PduA)-K89(PduJ), the closest distance measured between Cα atoms is 28.7 Å when homohexamers are assembled together following the disposition adopted in the 3NGK PduA crystal. Therefore, we believe that Trettel *et al* made a mistake in classifying this couple within the > 30 Å group in their figure 6D. The distance becomes 21.7 Å when considering the side-chain amine nitrogen atoms, which is the value that we indicated in our Figure 6A.

Therefore, it is not possible to reconcile our figures without first pointing out for instance this error. In our opinion, it would lead to a very long explanation and possibly to an irrelevant controversy, as the same conclusion prevails: several crosslinking reactions do not seem possible if only homohexamers composed these assemblies.

We tried to improve the explanation, both on the main text and figure captions (visible in the version with track changes). We hope the reviewer will approve the modifications.

*2. Page 24 "constitutive expression of Kpe grm2 in the gut, whereas the PDU or EUT would provide novel metabolic opportunities to the organism under inflammatory contexts"

This seems unlikely, as the authors point out themselves, Lactobacillus brevis is just one of the many non pathogens which have Pdu and Eut operons. 1,2-propanediol is produced by several intestinal organisms from fucose, ethanolamine derives from cell membranes in the diet and turned over in the intestine, they are both readily available for commensal organisms in the intestine. There is no Tetrathionate reductase in Klebsiella for the accelerated respiration of these compounds. The speculation to be made could be - exactly where in the intestine are choline and 1,2-PD , or choline and ethanolamine likely to be ? and does that correspond with K.pneumoniae carriage ?*

We fully agree with the reviewer that tetrathionate is unlikely to serve as alternative electron acceptor. We note, however, that other chemicals might serve as alternative acceptors. For instance, some authors proposed reduction of dietary Fe(III) (see reference 15).

But the reviewer might be right. Our sentence is too speculative, and probably unjustified. We decided to remove the commentary, as the topic is pretty complex and our expertise insufficient.

As a consequence, two references were removed from the final revised version.

*3. Typos etc Page 20 Line 11 "floopy" for floppy, "flexible" would probably be a better word here anyway.*

“Flexible” is now used in the revised version. *In a couple of places there are what look like direct translations P15 "all GRM2 BMC-H combinations conducted to strong fluorescence", P20 "Trettel’s et al argumentation*

*References
1. Trettel, D. S., W. Resager, B. M. Ueberheide, C. C. Jenkins, and W. C. Winkler. 2022. Chemical probing provides insight into the native assembly state of a bacterial microcompartment. Structure 30: 537-550.e5. "*

We are not sure to understand. Is the reviewer meaning that “conducted” is an inappropriate term, while “Trettel’s et al argumentation” is wrong and should instead be “Trettel *et al.*’s argumentation”?

We modified the two points anyway. We hope we understood correctly and things are better now.

*Reviewer #3: recommendation: accept following revision

L. Barth et al. report here on potential variations in the assembly of bacterial microcompartments (BMCs). BMCs are essential for the sequestration of biochemicals producing toxic or highly reactive intermediates, and are canonically considered to be assembled from a single set of protein shell components per associated reaction pathway. In this manuscript, genetic analysis of operons in K. pneumoniae identifies multiple homologous BMC-H genes and multiple types of BMC in a single organism, challenging this assumption. The authors use multiple orthogonal techniques, including in vivo, in vitro, and in silico assays, to assess the combinatorial formation of heterologous BMCs. While the conclusions reflect the somewhat messy and difficult to interpret nature of the complicated system, they are appropriately analyzed, reported accurately, and generally properly justified.

notable strengths
The heart of the paper appears to be figures 4 and 5, which are thoroughly analyzed. Possible caveats with respect to expression, tag interference in folding, and differentiating lack of interaction due to poor behavior from bona fide lack of interaction are appropriately addressed.

notable weaknesses
Details on statistical analysis are scant, but this should be easily correctable and seems unlikely to affect the conclusions of the manuscript.*

We now provide some additional details (see below).

*Conclusions based on structural prediction are not fully supported*

Globally speaking, both AF2 and ESMFold classified a majority of combinations as feasible, in good agreement with tGFP readings and other experimental observations. However, we agree that conclusions are less clear for a minority of cases, where discrepancies with experimental trends manifested too. We have tried to be more careful in raising conclusions in the new version. We hope we correctly figured out what the reviewer was referring to, since the commentary was not sufficiently precise.

*specific comments (sorted by figure)

Figure 1 - none

Table 1 and associated supplemental -
Major point: Please provide details of the statistical analysis. In particular, is each triplicate in table S1 treated separately or is ANOVA used within each gene's set of conditions? Ideally, it will be the latter.*

Quantifications were performed with the BioRad CFX Maestro software. In our analysis, the expression values were given as relative quantity to the condition without substrate (control sample) by setting the expression level of the control sample to 1. In this situation, the software normalizes the relative quantities of all target gene expression to the control quantity (a value of 1). The expression values are thus given as fold changes (relative quantity) relative to the condition without substrate (S1 Table).

In our experiment, each condition comprises 3 biological replicates and corresponds to a biological group. The expressions values reported in Table 1 were calculated from those of the 3 independent biological replicates by displaying the average expression of the samples within the biological group. Because expression is typically log-normally distributed, the expression is averaged using the geometric mean:

Where:

- Exp_1_, Exp_2_, Exp_n_= Relative quantity of the samples in the biological group

- n = Number of samples in the biological group (3 in our case)

In Table S1, the error bars values displayed for biological groups are based on the standard error of the mean (SEM) of the log_2_ of the expression. The error bars are calculated as follows:

Exp. Lower Error Bar = 2^Exp.(lg)-SEM Exp.(lg)^

Exp. Upper Error Bar = 2^Exp.(lg)+SEM Exp.(lg)^

Where:

- Exp.(lg) = log_2_ of the expression (normalized expression) for the biological group

- SEM Exp.(lg) = standard error of the mean of the expression (log_2_)

The P-values reported in S1 table are the results of unpaired t-tests comparing the distributions of per well Normalized Expression (NE) values for the control sample versus the test sample.

We believe this explanation is exceedingly long and too technical. But we understand reviewer’s concerns, and for that reason we added a more detailed explanation in the corresponding paragraph of the M&M section. We hope it will be helpful.

*Minor point: use of comma or period to indicate the decimal point is inconsistent (and in the supplemental tables in general)*

We did not realize when submitting the manuscript about this inconsistency, and we thank the reviewer for helping us to improve the presentation. The problem has been solved in the revised Tables.

We also profited to modify some other “formatting” errors. *Figure 2 and associated supplemental -
Major point: Related to Table S5, it is difficult to assess the reliability of the SEC analysis without seeing the trace itself. Is a clean peak seen, or is the highest part of a broad spread reported? Please provide in supplemental data. Caveats about certain samples appear to be appropriately noted in the text.*

The reviewer is right, the behavior might be easier to understand by looking at corresponding chromatograms. We added a two-panel figure (S3A and S3B Figs) with SEC-HPLC chromatograms for all investigated homo- and heteromeric samples.

As a consequence, all subsequent supplementary figures had to be renumbered.

*Minor point: according to methods, I believe these proteins had urea removed by dialysis to refold them before SEC analysis? It may be worth briefly noting this in the main text or appropriate legend.*

In fact, disassembled BMC-H from urea-treated samples were repurified on TALON columns. After several washes before the elution step with 300 mM imidazole, urea should be absent. The dialysis step was mostly intended to reduce the presence of imidazole and other buffer components that result in dominant peaks in HPLC profiles (eluting at 9-11 mL volumes). These species were still visible at low levels in many chromatograms. Sometimes we also injected the proteins after purification, without carrying the dialysis step, with similar results in terms of the elution time/volume of protein peaks.

We modified a sentence in the main text to further clarify this point (page 10: Corresponding purified fractions, thus in the absence of urea, remained soluble.) *Figure 3 -
Minor point: Ordering of panels by letter is odd.
Minor point: It would be helpful to provide an image of at least one of the constructs that failed to assemble as a negative result against which to compare the less evident assemblies such as panels B and F.*

An image from a negative case (EutK, panel C) is included now.

We understand reviewer’s claims with regard to panel F. With the intention to provide further evidence, we included a second panel for this case. Yet, evidenced objects are hard to see, they sometimes appear as thin stripes. Unfortunately, they are less evident in uploaded figures than in raw images.

On the contrary, panel B is relatively clear in our hands. We provide anyway a slightly higher magnification view of the same objects in the revised version. We hope motifs are clearer now?

In fact, panel order was function of image size, as unfortunately format changed over the course of this study as a consequence of a spoilt detector, which had to be replaced by a newer version. We tried now to rearrange in a better manner the panels. *Figure 4 -
Figure S3 is helpful in understanding the experiment.
Minor point: Was tripartite GFP chosen instead of split GFP to minimize the size of the tag attached to the individual BMC constructs?*

Yes, we were concerned by potential interferences with oligomerization and even for formation of higher-order assemblies. For instance, a small SUMO domain in fusion to several BMC-H was shown to prevent hexamer assembly, something that was exploited to augment protein concentrations before triggering shell formation by proteolytic release of such domains (Hagen et al., *In Vitro Assembly of Diverse Bacterial Microcompartment Shell Architectures*, Nano Lett. 2018). We therefore assumed that the relatively big split GFP partner (β-strands 1 to 10) might have similar consequences. *Figure 5 -
Major point - as with Table S1, please provide details of statistical analysis*

We have now included the next commentary in the figure caption: Plotted mean F_max_ and standard deviations derive from at least 2 independent repetitions of experiments, each one performed on 3 clones per library member. These values are provided in supplementary File S1.

Numerical values of standard deviations were in fact provided in the Supplementary File S1. For instance, those presented in old Fig. 5A correspond to elements lying in the diagonal (part of them).

It is necessary to say that as a consequence of dividing these plots in two portions with the intention to better highlight differences among lowest fluorescent cases, the upper part became compressed, which might erroneously give the impression of extremely low SD for such cases. In an attempt to improve things, we included now the numerical value of the lowest Y-axis mark of such top representations in Figures 5 and S5 and S10 Figs. *Figure S5 -
Major point - I am not convinced that this supports the statement that these tags interfere somewhat with assembly formation, largely because so many of bands are barely visible to begin with (and why does EutK not appear to change in size?). Analysis may be best limited to confirmation of expression. Failure to observe expression of a construct by this method should be highlighted, but merely indicates that the protein is not abundant enough to be seen above lysate baseline; any noise in the data due to poor expression would already be addressed in Figure 5.*

We partly disagree.

Let us for instance consider data presented in Fig.2 for CmcA (His6). The protein is well expressed but totally absent from the soluble fraction. We demonstrated that this was due to the fact that the hexamer was retained forming higher-order assemblies, which were pelleted with cellular debris. Let us now consider the SDS-PAGE data presented in old Fig. S5 (S6 Fig in the revised version). Band intensities are practically identical in cellular and soluble fractions. As expression levels are considerably high for many constructs, for instance in the first lane for CmcA-GFP10, we conclude that such constructs are less prone to form higher-order assemblies.

With regard to EutK size, the reviewer is right. Unfortunately, we do not have a clear answer to this point. Expression plasmids were verified. Moreover, it is extremely unlikely that a mistake was committed on 4 different strains. The fact that PduK species were seen at different sizes indicates that EutK species should have been resolved too (both of them have considerably higher MW than all other BMC-H).

We note, however, that apparent MW differences induced by GFP10 or GFP11 tags seem overestimated, in view of tiny MW differences among them (< 0.2 kDa). Other physicochemical properties must therefore play. It is for instance known that anormal migration (gel shifting) occurs in many proteins as a consequence of imbalances in interactions with SDS molecules. This happens for hydrophobic proteins, also when high number of basic or acid residues are present (see for instance Shi Y *et al*, Abnormal SDS-PAGE migration of cytosolic proteins can identify domains and mechanisms that control surfactant binding, Prot Sci, **2012** Jun 12;21(8):1197–1209).

When looking EutK sequence, this argument does not seem really justified: 19 Asp+Glu residues, 17 Arg+Lys, theoretical pI of 5.7 calculated by the ProtParam tool. However, the distribution seems to be pretty different when inspecting separately the BMC-H core (pI 4.5), and the C-ter extension (pI 10.4). We therefore hypothesize that abnormal differences induced by GFP10 and GFP11 presence might fade away if the effect caused by the C-ter extension was dominant. However, this point was out of the scope of our study, and we did not pursue further the investigation.

*Figure 6 -
Appropriately analyzed and discussed in text
Minor point: Bringing the arrows in front of the lane dividers instead of behind, and perhaps making them brighter, may make the figure easier to interpret without zooming far in*

We tried to improve the representation but transferring the arrows in front of lane dividers, and not behind as reviewer demands. However, it was not possible to keep a common pattern for the totality of arrows since it would lead to covering some protein bands, as a consequence of lane-to-lane separations that fluctuate.

We hope the new representations of Fig 6 and S8 Fig (old Fig. S7) are now better. *Figure 7 -
No comments

Structural prediction -
Major point: discussion of c-terminal extensions depends on their reported local confidence, which given the predictions appears low. Either justify the accuracy of the predictions, possibly by coloring Figure S2 by local confidence, or remove discussion of extension structure from the manuscript.*

We understand reviewer’s concern. We added within S2 Fig a representation of pAE values provided by Alphafold, which should be helpful. The scores calculated between pairs of residues belonging to the same monomer (diagonal elements) support the existence of an additional domain in the C-terminal part of EuK, and possibly a much smaller one in PduK. In the case of CmcE, the C-terminal extensions is completely unstructured.

In fact, AF2 predictions can be directly recovered from the EMBL-EBI repository. Here below, two figures showing EutK (entry B5XVQ9, left) and PduK (entry B5XUS7, right) monomers:

The C-terminal domains are therefore considered as highly confident (pLDTT values between 70 and 90).

We therefore added this sentence at page 10 “*Indeed, high confidence AF2 scores (pLDTT between 70 and 90) are attributed to these portions in models deposited at the EMBL-EBI site (EutK entry B5XVQ9; PduK entry B5XUS7).*”.

On the other hand, we recall that such domains were characterized in a crystal structure of an EutK homolog (Tanaka S et al. Science, 2010) or proposed to explain biochemical data with *S. enterica* PduK (Crowley CS et al. JBC, 2010).

*Minor point: if pTM and ipTM values from AlphaFold-Multimer are available, please add to Table S3*

We introduced requested scores in the tables, though we believe it is mostly redundant with other scores. However, some cases might be better perceived now.
